# Widespread Genomic Incompatibilities in *Caenorhabditis elegans*

**DOI:** 10.1534/g3.114.013151

**Published:** 2014-08-15

**Authors:** L. Basten Snoek, Helen E. Orbidans, Jana J. Stastna, Aafke Aartse, Miriam Rodriguez, Joost A.G. Riksen, Jan E. Kammenga, Simon C. Harvey

**Affiliations:** * Laboratory of Nematology, Wageningen University, 6708 PB Wageningen, The Netherlands; † Biomolecular Research Group, School of Human and Life Sciences, Canterbury Christ Church University, North Holmes Road, Canterbury, CT1 1QU, UK

**Keywords:** *C. elegans*, Bateson-Dobzhansky-Muller, reproductive isolation, negative epistasis, QTL, introgression line

## Abstract

In the Bateson-Dobzhansky-Muller (BDM) model of speciation, incompatibilities emerge from the deleterious interactions between alleles that are neutral or advantageous in the original genetic backgrounds, *i.e*., negative epistatic effects. Within species such interactions are responsible for outbreeding depression and F2 (hybrid) breakdown. We sought to identify BDM incompatibilities in the nematode *Caenorhabditis elegans* by looking for genomic regions that disrupt egg laying; a complex, highly regulated, and coordinated phenotype. Investigation of introgression lines and recombinant inbred lines derived from the isolates CB4856 and N2 uncovered multiple incompatibility quantitative trait loci (QTL). These QTL produce a synthetic egg-laying defective phenotype not seen in CB4856 and N2 nor in other wild isolates. For two of the QTL regions, results are inconsistent with a model of pairwise interaction between two loci, suggesting that the incompatibilities are a consequence of complex interactions between multiple loci. Analysis of additional life history traits indicates that the QTL regions identified in these screens are associated with effects on other traits such as lifespan and reproduction, suggesting that the incompatibilities are likely to be deleterious. Taken together, these results indicate that numerous BDM incompatibilities that could contribute to reproductive isolation can be detected and mapped within *C. elegans*.

To understand the mechanisms that lead to speciation, insight is required into the genetic basis of reproductive isolation. The most widely accepted explanation for the genetic basis of intrinsic, postzygotic reproductive isolation between species is the Bateson-Dobzhansky-Muller (BDM) model (Bateson 1909; Dobzhansky 1936; Muller 1942). This relies on negative epistasis between alleles and normally considers the case of alleles that have been fixed in different lineages. In hybrids, negative epistasis between alleles that have not been tested together by natural selection result in reduced hybrid fitness (Phillips 2008). Such epistatic interactions have been shown to be involved in, for instance, hybrid male sterility in *Drosophila* (Perez and Wu 1995; Orr and Irving 2001; Tao *et al.* 2003) and are also important in human disease and in complex traits more generally (see Phillips 2008 and Mackay 2014 for review). In recent years, the causal polymorphisms underlying BDM incompatibilities have been identified in a limited number of species, with divergence in both coding sequence and in regulatory elements producing incompatibilities (see Presgraves 2010 for review).

BDM incompatibilities will, however, also arise within species (see Cutter 2012 for review) and theoretical analyses suggest that interactions between synthetic deleterious loci are common (Phillips and Johnson 1998; Lachance *et al.* 2011). This is supported by the widespread observation of outbreeding depression in hybrids between divergent populations (*e.g.*, Templeton 1986; Edmands 1999; Dolgin *et al.* 2007; Drury *et al.* 2013; Gimond *et al.* 2013). A small number of BDM incompatibilities have now been identified within species, mostly producing major effects (*e.g.*, Seidel *et al.* 2008; Bikard *et al.* 2009; Drury *et al.* 2011; Baird and Stonesifer 2012). More recently, a genome-wide screen in *D. melanogaster* recombinant inbred lines (RILs) identified many epistatic interactions, two of which were shown to have major effects on fecundity (Corbett-Detig *et al.* 2013). It is, however, likely that the alleles and regions that have been found to date represent only a subset of the polymorphic incompatibilities within a species, *i.e.*, the major effects identified to date represent those that are easy to detect (see Rockman 2012 for a general discussion of this issue).

As outbreeding depression has been documented between isolates of the free-living nematode *Caenorhabditis elegans* (Dolgin *et al.* 2007) it is likely that a range of potential incompatibilities exists between isolates. We therefore sought to identify small-effect incompatibilities between the isolates CB4856 and N2. We sought these by looking at the disruption of a complex, highly regulated and coordinated, phenotype, egg-laying, and undertook screens for genomic regions that disrupt this process. At 20°, *C. elegans* N2 eggs are normally laid about 3 hr after fertilization at around the 30-cell stage (Hirsh *et al.* 1976), with hatching occurring approximately 14 hr later (Sulston *et al.* 1983). Disruption of the egg-laying process produces an *egl* (*eg*g *l*aying abnormal) phenotype, with one class of *egl* mutation characterized by an increase in the number of fertilized eggs retained within the body and eggs being laid at a much later stage of development. Mutations producing this *egl* phenotype have been identified in genes that affect chemosensation, muscle development, the cell lineage, sex determination and dauer larvae development (Greenwald and Horvitz 1980; Horvitz and Sulston 1980; Waterston *et al.* 1980; Trent *et al.* 1983; WormBase [www.wormbase.org]). We therefore considered that this phenotype represented a suitably large target for the development of incompatibilities. Screens were undertaken using *C. elegans* RILs and introgression lines (ILs) produced from the isolates CB4856 and N2 (see the section *Materials and Methods* for details of these lines) and identified multiple quantitative trait loci (QTL) that result in a synthetic *egl* phenotype. For two of the QTL regions identified, analysis of the ILs indicates that the incompatibilities are a consequence of complex interactions between multiple loci. Incompatibility regions identified in these screens are also shown to be associated with negative effects on lifespan and on reproduction, suggesting that the incompatibilities are likely to be deleterious. In combination, these results indicate that numerous BDM incompatibilities that could lead to reproductive isolation can be detected within *C. elegans*.

## Materials and Methods

### Worms

Experiments were performed using the N2 (Bristol) isolate (obtained from the *Caenorhabditis* genetics center), wild isolates of *C. elegans* (obtained from Marie-Anne Félix, IBENS, Paris, France, and from the CGC), RILs produced from crosses between CB4856 and N2 (see, for details, Li *et al.* 2006; Kammenga *et al.* 2007, 2008; Li *et al.* 2010; Viñuela *et al.* 2010; Elvin *et al.* 2011; Rodriguez *et al.* 2012; Viñuela *et al.* 2012), and a panel of CB4856/N2 ILs derived from these RILs in which regions of the CB4846 genome have been introgressed into an N2 background (see, for details, Doroszuk *et al.* 2009; Green *et al.* 2013). Briefly, the RILs were created from crosses between N2 and CB4856, with the F1 progeny subsequently inbred, by transfer of single animals at each generation, for 20 generations. RILs were then genotyped at 121 markers across the genome (20 each on chromosomes *I*, *II*, *III*, *IV and X*, and *21* on *V*). The ILs were produced from specific RILs, chosen based on the CB4856 regions they contain, these RILs were back-crossed to N2, genotyped, further back-crossed as appropriate, and then genotyped at the same markers as the RILs and at two additional markers on chromosome *IV* (for a total of 123 markers). This resulted in the production of a panel of ILs, each containing a single segment of the CB4856 genome in an N2 background.

Worms were maintained using standard methods and fed on the OP50 strain of *Escherichia coli* (Stiernagle 2006). All experiments were undertaken at 20° and were initiated from synchronized populations of L1s produced by allowing eggs isolated from hypochlorite treated adults (Stiernagle 2006) to hatch on plates without food and to develop for 24 hr. Within assays, genotypes were randomized and plates blind coded, with plates that became infected by fungi excluded from analyses.

### Embryo stage analysis in the RILs and ILs

The various stages of embryo morphogenesis are well defined in *C. elegans* (Von Ehrenstein and Schierenberg 1980) and can be identified with a dissecting microscope. Most screens undertaken for mutations producing an *egl* phenotype relied on screening worms early in the reproductive period to identify hermaphrodites that had died by internal hatching of progeny (bagging) or that were bloated with late-stage eggs (Greenwald and Horvitz 1980; Horvitz and Sulston 1980; Waterston *et al.* 1980; Trent *et al.* 1983). Subsequent analysis of these mutants showed that most worms capable of releasing eggs tended to lay them at a much later stage of development than the wild-type (Trent *et al.* 1983). As we aimed to identify genomic regions that, when in a different genetic background, produced an *egl* phenotype, we determined the stages of eggs laid by worms late in the reproductive period. Our reasoning for screening late in the reproductive life is that this would allow the identification of differences reliant on age-related loss-of-function. Preliminary experiments (data not shown, but see Figure 4 and Figure 5) indicated that both N2 and CB4856 continue to lay almost all eggs at very early stages of development (Supporting Information, Figure S4) throughout the reproductive period. We therefore considered that laying eggs at a late stage of development could be considered a consequence of an incompatibility between N2 and CB4856 alleles.

For embryo stage analysis, we classified progeny into four stages: stage I from fertilization to the end of gastrulation; stage II from ‘lima bean’ to ‘comma’ stage embryos; stage III ‘tadpole’ to ‘pretzel’ stage; and L1 (stages as described by Von Ehrenstein and Schierenberg 1980; see also Trent *et al.* 1983). Unless otherwise noted, embryo stages were assayed on the third day of reproduction, 6 d after recovery from L1 arrest, with adults transferred to fresh NGM plates 5 d after feeding to allow progeny to be discarded. On the day of assay, for each genotype, 5−10 worms were moved to a fresh NGM plate for 2 hr and then discarded. Eggs laid within this 2-hr window were then observed and the developmental stage classified. For the RIL and IL assays, lines were randomized across experimental blocks and N2 and CB4856 wild types were included as controls in each block. Other assays were conducted in the same manner. Analysis of embryo staging for each experimental block took less than an hour, and rescoring of plates during initial experiments indicated that this time did not affect embryo stage data.

All analyses were conducted in custom written scripts in “R” version 2.13.1 × 64. To analyze these data, the effect of genotype on the stage at which the eggs were deposited was tested by analysis of variance, with all the individual egg stage scores used as input “egg-stage~genotype+e.” This was only used to determine the effect of the genotype on the variation in egg-stage. For the IL and RIL data, the mean square of the trait and the residuals were then used to determine the heritability of the trait in each panel. To find genomic regions associated with the control of egg stage, we used QTL mapping. For QTL mapping in the RILs, we used a single marker model, with the percentage of total progeny at a certain stage used as a phenotype. In the RILs, the percentage of progeny at > stage II also was mapped. Genome-wide thresholds were determined by 1000 permutations. In each permutation round, the phenotypic scores were distributed randomly over the RILs after which genome-wide QTL were mapped. The most significant linkage was recorded for each permutation round. The 95% highest –log10(p) value was taken as the 0.05 genome-wide threshold. A similar method was used to determine the threshold for multiple QTL mapping (MQM).

### Bin mapping

Bin mapping in the ILs was done as described by Doroszuk *et al.* (2009) and Green *et al.* (2013), with the exception that a χ^2^ test was used as a statistical test. The percentages of eggs per stage of N2 was used as expected distribution and tested against the distribution per bin. Threshold was determined by 10,000 permutations. Each permutation picked the egg-stage scores of two groups of three randomly selected dishes. These two groups were then used in a χ^2^ test. The 95% highest –log10(p) value was taken as the 0.05 FDR threshold. This method was also used to determine the threshold in IL *vs.* IL mapping.

### MQM method

A forward marker selection was used as MQM method. The mapping was initiated by single marker mapping. The marker with most significant linkage was added to the mapping model as a cofactor. The cofactor was excluded from the model when markers closer than five markers from the cofactor were considered or when the significance of the cofactor was > 0.05. This process was repeated until no new QTL/cofactors could be added.

### Fixed locus mapping

To investigate the effect of the major QTL of the left of chr IV on QTL mapping, we fixed the locus by splitting the RILs into two groups. One group with an N2 allele at the left of chr IV and one group with a CB4856 allele at the left of chr IV. Single marker mapping using linear regression was subsequently used to find QTL in these two groups of RILs.

### Sub-IL generation

To further investigate the effects of introgressions on chromosome IV on the control of egg stage, we also analyzed an additional set of sub-ILs (ewIR4001-4011). These were generated by crossing ewIR052 with N2 and selecting for new recombinants in the F4 offspring. F4 offspring were obtained by single worm decent. Restriction fragment length polymorphism markers described in Li *et al.* (2006) and Doroszuk *et al.* (2009) spanning the original ewIR052 introgression were used for recombination detection.

### IL *vs.* IL mapping

To test whether the egg-stage distribution between each IL-pair were different, a χ2 test was used. The percentages of eggs per stage of one IL was used as expected distribution and tested against the distribution of the other IL. Pairs were then compared as described by Shao *et al.* (2010) and Green *et al.* (2013) to find QTL.

### Embryo stage analysis in wild isolates

Preliminary experiments and the RIL and IL analyses indicated that N2 and CB4856 lay the majority of their eggs at very early stages of development. To investigate natural variation in this trait more broadly, we assayed, as described previously, a range of wild isolates. The IL ewIR51, which contains a CB4856 introgression on chromosome IV that results in the production of large numbers of late stage progeny (see Figure 2 and Figure S2), was included in these assays as a control.

### Analysis of the chromosome IV QTL

To determine how the embryo stage of progeny changed across the reproductive period, we compared ewIR51, ewIR52 (another IL containing the chromosome IV QTL), CB4856, and N2. Here the embryo stages of progeny were determined, as described previously, daily for the first 3 d of the reproductive period. To determine whether the production of large numbers of late stage embryos was associated with an increase in the number of fertilized eggs *in utero*, as seen in many *egl* mutants (Trent *et al.* 1983), we compared ewIR51, ewIR52, and N2. To do this, individual hermaphrodites were transferred to a drop of hypochlorite solution (Stiernagle 2006) on an NGM plate with food. Plates were then incubated at 20° for 2 d when the number of progeny that had developed was determined. Again, these assays were undertaken daily for the first 3 d of the reproductive period.

### Relationship to other traits

To determine how variation in other life history traits relates to the synthetic *egl* effects observed in the RIL and IL lines, all ILs containing introgressions on chromosomes II and IV were assayed for body length, lifetime fecundity and lifespan. These analyses also identified any animals that died by bagging. These assays used standard methods for the analysis of reproductive traits in *C. elegans* (Hodgkin and Doniach 1997). Body length was determined as described by Harvey and Orbidans (2011) for worms 2 d after recovery from L1 arrest, with individuals photographed using a Moticam 2000 video camera (Motic, Wetzlar, Germany) and the length from the mouth to the base of the tail, determined in ImageJ (http://rsb.info.nih.gov/ij/). Worms were considered to have died if they were not moving and failed to respond to touch.

### Data storage

All data were stored in WormQTL (www.wormqtl.org; Snoek *et al.* 2013, 2014b; Van Der Velde *et al.* 2014).

## Results

Analyses of the RILs (101 lines) and the ILs (87 lines) indicated that genotype significantly affected the stage at which eggs were laid (*P* < 1e-15 in both cases). The heritability of the egg stage was also very high (estimated as 96.1% in the RILs and 92.9% in the ILs based on individual egg measurements and 74.8% on multiple population averages per genotype in the ILs), although variability between replicates suggests that the heritability based on the individual egg measurements is most likely an overestimation. In both sets of lines, the N2 and CB4856 controls are not significantly different (χ^2^ test; p~1), with both lines laying mostly stage I eggs (~96% and ~90%, respectively, for N2 and CB4856).

**Figure 1:**
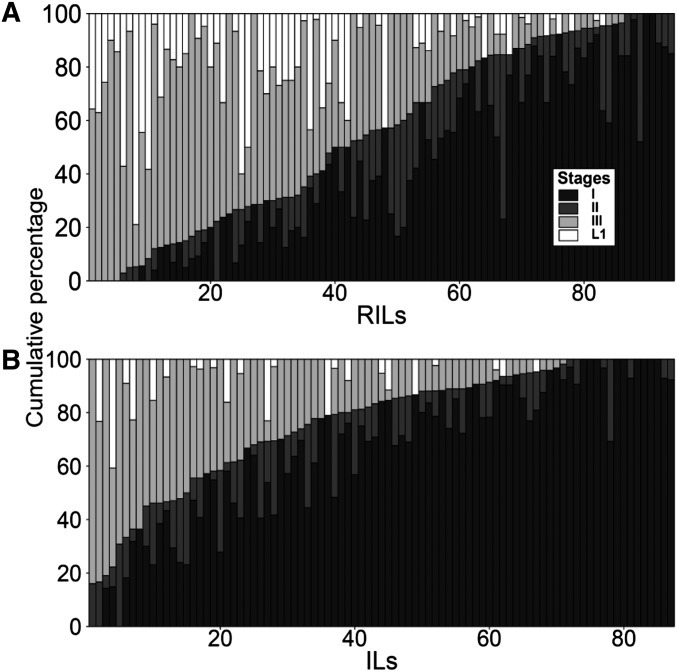
Embryo stage distribution in the RILs and ILs. The cumulative percentage of embryo stages per RIL (A) and IL (B). Lines were sorted by the percentage of embryos > stage II. RILs, recombinant inbred lines; ILs, introgression lines.

The phenotypic distribution in both the RILs and ILs shows a one-sided transgression, with many genotypes laying large proportions of their eggs at much later stages (Figure 1 and Figure S1) than either of the parental isolates. About half of the RILs laid 50% or more eggs at stage III or later, with about 20% of the ILs displaying such extreme phenotypes (Figure 1). These lines therefore phenocopy mild *egl* mutations, *i.e.*, they would be classified as M/E, most/early, with all or most progeny released, a few early-stage eggs, and many late-stage eggs observed on the plate (Trent *et al.* 1983). The observed transgression therefore provides evidence that the stage at which an egg is deposited is a polygenic trait. Moreover, it suggests that either N2 or CB4856 each carry positive and negative allele(s) of the genes involved that are acting additively, or that the observed effects are a consequence of incompatibilities between diverged N2 and CB4856 alleles at different loci, *i.e.*, negative epistatic effects, or a combination of both of these. That more RILs than ILs show an *egl* phenotype, suggests that multiple regions of the genome and interactions between those contribute to the laying of late stage eggs (comparison between Figure 1, A and B).

### QTL mapping in the RILs and ILs

**Figure 2:**
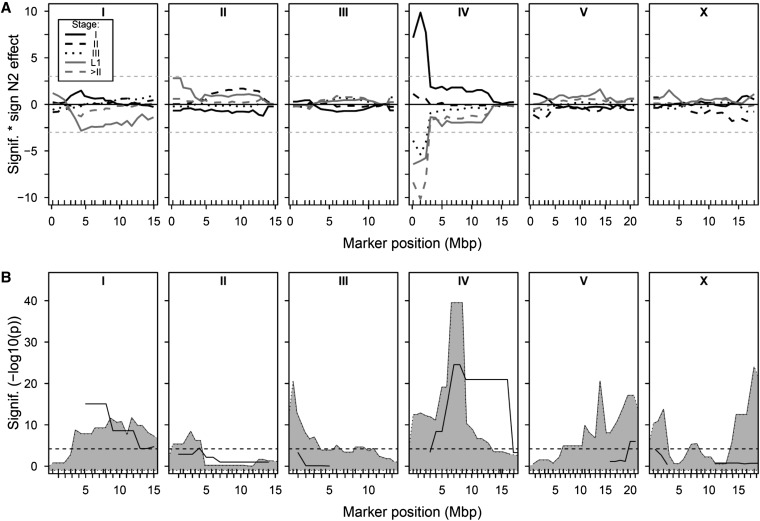
QTL mapping in the RILs and ILs. (A) Mapping of embryo stage in the RILs, with the significance (−log10(p)) multiplied by the sign of the effect of the N2 allele plotted against the marker positions in mega base pairs for the percentage of total eggs in stage I eggs (black solid line), stage II eggs (black dashed line), stage III eggs (black dotted line), stage IV eggs (gray solid line), L1s (gray dashed line), and the proportion of progeny > stage II (gray dotted line). (B) Genome-wide bin mapping of late-stage embryo production (proportion > stage II), showing the significance (−log10(*p*)) by chi-square test of ILs sharing a certain genomic part against N2. RILs, recombinant inbred lines; ILs, introgression lines.

QTL mapping in the RILs identified one highly significant locus at the left of chromosome IV (Figure 2A). This locus can be found for the percentage of progeny at stage I, stage III, L1, and > stage II, with the CB4856 allele at this locus increased the proportions of late stage progeny. These analyses also identified minor QTL for the proportion of L1s on both chromosomes I and II. MQM analysis indicated that additional QTL can be detected on chromosomes I, III, and IV (Table 1 and Figure S2). A two-locus scan for epistatic interactions suggested that there were interactions between many of these QTL, but, due to limited power, these were not significant after correction for multiple testing.

**Table 1:** Locations and effect of QTL detected for egg-stages

Chr	N2L	CBL	CBR	N2R	CB Effect	Detected by
I	2818974	3502476	3502476	4338254	+	MQM, (BIN), Single IL, IL vs. IL
I	9569913	10259909	10259909	11085295	+	Single IL, IL vs. IL
I	11085295	11085295	11085295	11760179	−	IL vs. IL
II	2755074	3403575	4147051	4800868	+	(BIN), Single IL, IL vs. IL
II	4147051	4800868	10414073	11180836	−	IL vs. IL
III	5925983	6847169	7998164	8318553	−	IL vs. IL
III	10027496	10613119	10613119	11341120	+	MQM, Single IL, IL vs. IL
III	10613119	11341120	11341120	12301725	−	IL vs. IL
IV	Not applicable	151889	1381409	2288742	+	SM, MQM, Single IL, IL vs. IL
IV	2288742	3067374	3067374	3920366	+	SM, MQM, Single IL, IL vs. IL
IV	10122930	10909560	10909560	11668242	−	IL vs. IL
IV	10909560	11668242	11668242	12748880	+	SM, MQM, Single IL, IL vs. IL
V	10368660	10912994	16008404	17377158	+	Single IL, IL vs. IL
V	17377158	18574593	18574593	19525561	−	IL vs. IL
V	18574593	19525561	20758352	20893784	+	Single IL, IL vs. IL
X	5010049	5770179	5770179	7067019	−	IL vs. IL
X	5770179	7067019	7982354	8691677	+	Single IL, IL vs. IL

The column label Chr show the chromosome on which the QTL was found. N2L, CBL, CBR, and N2R show the position of the left N2, left CB, right CB, and right N2 boundaries of the QTL. The “Detected by” indicates the methods by which the QTL were found/supported. QTL, quantitative trait loci.

Bin mapping in the ILs using the data from the initial genome-wide screen identified a total of 8 QTL where the CB4856 introgression increased the production of late-stage eggs (Figure 2B). ILs with introgressions harboring one of these QTL were retested in a separate experiment and this analysis resulted in the confirmation of four of the eight QTL (Table 1 and Figure 2B), with three of these QTL overlapping the major QTL and minor QTL identified in the RILs. The IL analysis also suggests the presence of additional QTL on chromosome V and on the X chromosome. In combination, the RIL and IL analyses therefore reproducibly identify regions of chromosomes I, II and IV where introgression of the CB4856 region into an N2 background results in an increased production of late-stage eggs.

**Figure 3:**
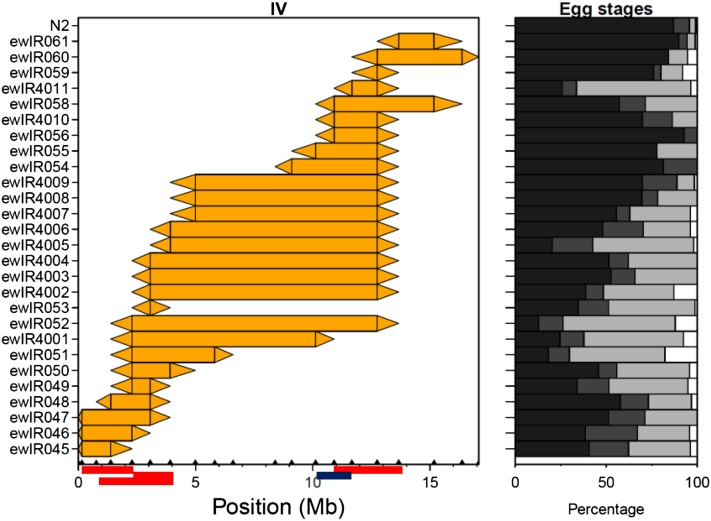
Comparison between ILs of chromosome IV. The CB4856 introgression per IL is shown by the colored rectangle. Triangles join adjacent CB4856 and N2 markers. Embryo stage distribution is shown as cumulative percentage of total progeny. From dark to light: Stage I, II, III, and L1 (in white). QTL are indicated on the X axis by red (+) or blue (−) boxes (denoting that the CB4856 allele increases or decreases the proportion of late stage embryos, respectively). ILs, introgression lines; QTL, quantitative trait loci.

The QTL identified by bin mapping span very large regions of the genome, up to almost a whole chromosome in the case of chromosomes I and IV (Table 1 and Figure 2B). Because of this, we investigated the individual ILs for clues on the number of alleles/QTL present. This was done by using a χ^2^ test to test for a difference in stage numbers between N2 and the individual ILs (Table 1 and Figure S3). These analyses detect and confirm the stage increasing CB4856 QTL on chromosomes I, II, III, and IV. Given that ~90% of progeny in N2 and CB4856 are stage I eggs, comparison of the ILs and N2 will only detect CB4856 alleles that increase progeny stage. Such analyses suggest that many regions of the genome disrupt the normal process of egg-laying. For example, on chromosome I this suggests the presence of at least three separate QTL as three nonoverlapping ILs are different from N2 (Table 1, Table S1 and Figure S3). In contrast to such analyses, comparison of overlapping ILs allows the identification of regions that contain CB4856 alleles that decrease progeny stage (Table 1 and Figure 3). These comparisons support the conclusions that the QTL detected here can be separated into multiple factors.

### Embryo stage analysis in wild isolates

**Figure 4:**
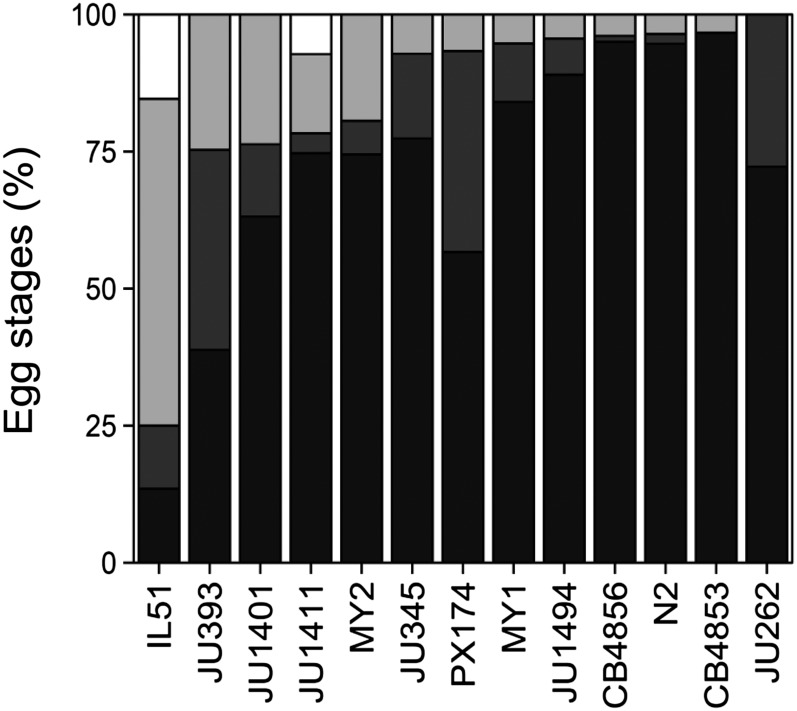
Embryo stages of wild-isolates. Embryo stage distribution shown as cumulative percentage of total progeny. From dark to light: Stage I, II, III, and L1 (in white). CB4856 scores from different experiments (n = 281). (N = IL51 (ewIR51): 52; JU393: 85; JU1401: 76; JU1411: 83; MY2: 98; JU345: 84; PX174: 30; MY1: 94; JU1494: 91; CB4856: 103; N2: 112; CB4853: 60; JU262: 18). For > stage II eggs all the wild isolates are significantly different from ewIR51 (*P* < 0.01, two-sided *t*-test on plate averages). None of the > stage II differences between the wild-isolated were significantly different (*P* > 0.05, two-sided *t*-test on plate averages).

To determine whether late-stage egg production was seen in wild isolates of *C. elegans*, the embryo stage of hermaphrodites from a range of wild isolates on the third day of reproduction was tested. These analyses indicated that there are differences between lines, but that wild isolates all lay eggs at a predominantly early stage of development (Figure 4). This further supports our classification of the late-stage embryo production trait as an incompatibility.

### Analysis of the chromosome IV QTL

**Figure 5:**
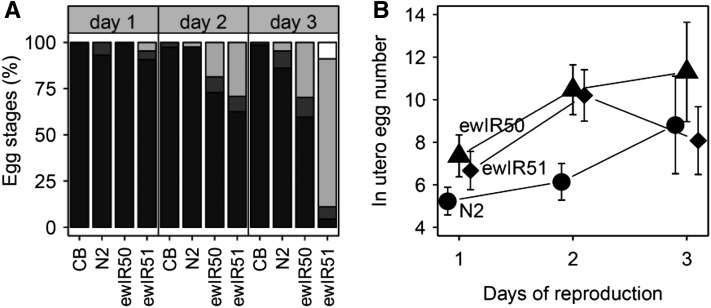
Variation across the reproductive period. (A) Embryo stage distribution across the reproductive period shown as cumulative percentage of total progeny. From dark to light: Stage I, II, III, and L1 (in white). (B) Number of eggs *in utero* across the reproductive period.

Analysis of embryo stage across the reproductive period indicates that the trait is age-related, such that the proportion of embryos laid at later stages of development increases throughout the reproductive period (Figure 5A). This finding suggests that it may represent a change in the rates at which the worms are senescing. Previously identified differences in developmental speed between RILs derived from crosses between the isolates N2 and CB4856 only span a few hours (Francesconi and Lehner 2014; Snoek *et al.* 2014a) and cannot therefore cause the (large) differences in egg-stages between lines.

Many *egl* mutations cause worms to retain large numbers of eggs *in utero*, with young adults displaying a slightly bloated phenotype and older worms often containing many times the normal number of fertilized embryos. Comparison of two ILs containing the major chromosome IV QTL to N2 (Figure 5B) indicated that the number of eggs *in utero* is slightly increased during the first two days of reproduction, but that there is no increase seen on the third day of reproduction.

### Relationship to other traits

**Figure 6:**
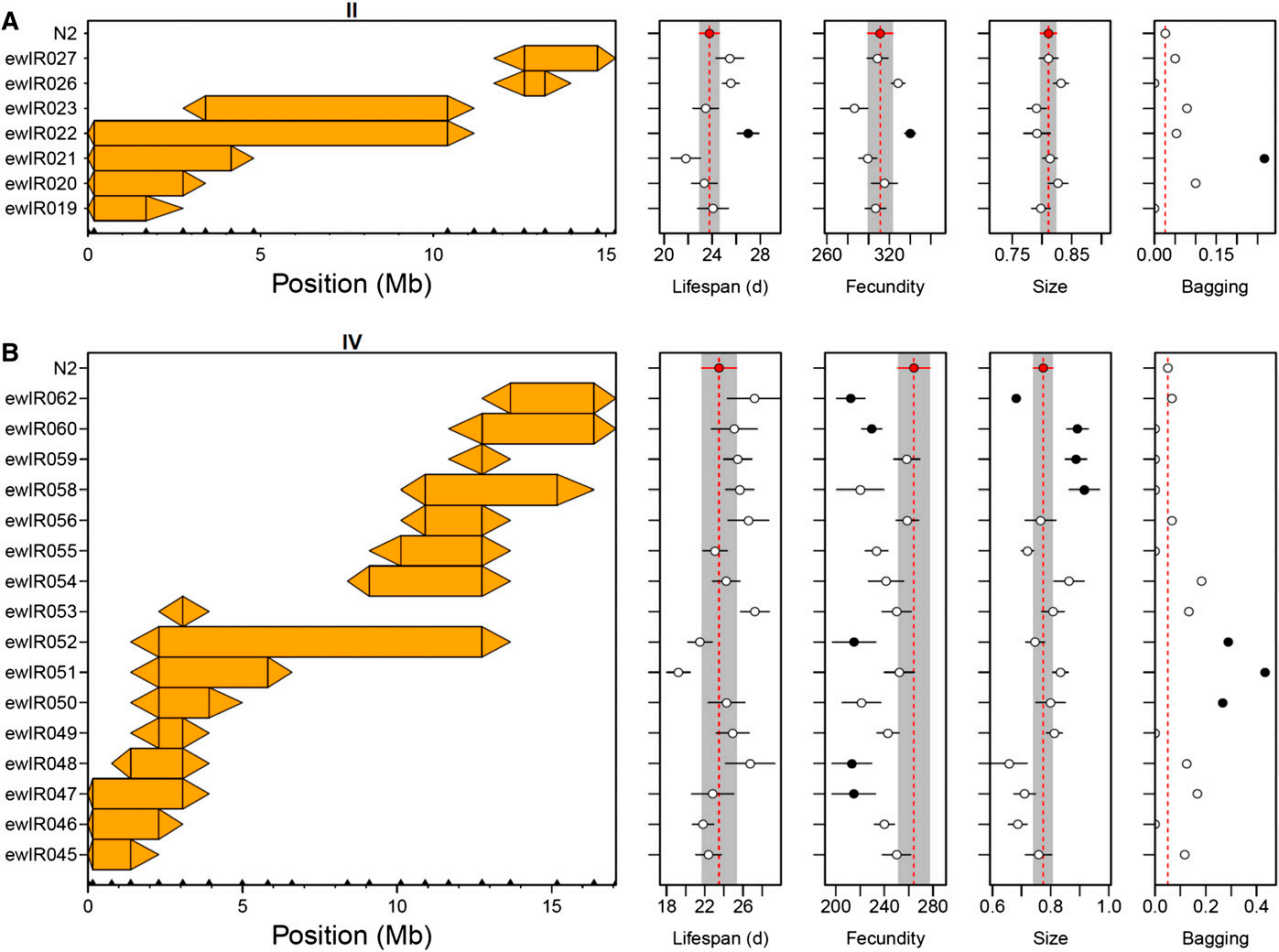
Incompatibility QTL are associated with variation in other traits. Average lifespan, lifetime fecundity, body size at L4 and proportion of worms that die by bagging for ILs containing introgressions on chromosome II (A) and IV (B). The CB4856 introgression per IL is shown by the colored rectangle. Triangles join adjacent CB4856 and N2 markers. Error bars represent ± 1 SE, dashed lines and shaded bars represent trait values in N2 and ILs significantly different from N2 (*P* < 0.05) are shown in black. QTL, quantitative trait loci.

Analysis of all ILs containing introgressions on chromosomes II and IV indicated that all traits were variable (Figure 6, A and B), with these analyses defining QTL for all traits (Table 2 and Table S2). Comparison of these QTL to those found in previous analyses indicates that many QTL are found in multiple studies. For instance, variation in body size between N2 and BO has previously been mapped to chromosome IV (Knight *et al.* 2001), and one of the chromosome IV body size QTL identified here (Table 2) contains *tra-3*, a gene polymorphic between CB4856 and N2 that affects how body size changes across temperatures (Kammenga *et al.* 2007). Similarly, previous comparisons using CB4856 and N2 RILs identified a fecundity QTL on chromosome IV (Gutteling *et al.* 2007), although this was found at 12° and not at 24°. The patterns of variation identified here do however indicate that the control of these traits is complex, with chromosome IV containing five separate QTL affecting body size (Table 2).

**Table 2:** Locations and effect of QTL detected for body length, lifetime fecundity, lifespan, and bagging

Trait	Chr	N2L	CBL	CBR	N2R	CB effect	Detected by
Size	IV	766649	1381409	3067374	3920366	−	Single IL
	IV	5819735	6599685	12748880	13667267	−	IL *vs.* IL
	IV	8397264	9102404	9102404	10122930	+	IL *vs.* IL
	IV	11668242	12748880	12748880	13667267	+	Single IL
	IV	12748880	13667267	16371991	17084259	−	Single IL
Lifespan	II	Not applicable	176721	2755074	3403575	+	IL *vs.* IL
	II	4147051	4800868	10414073	11180836	+	IL *vs.* IL
	IV	3920366	4991858	5819735	6599685	−	IL *vs.* IL
Fecundity	II	Not applicable	176721	2755074	3403575	+	IL *vs.* IL
	II	4147051	4800868	10414073	11180836	+	IL *vs.* IL
	IV	12748880	13667267	16371991	17084259	−	Single IL, IL *vs.* IL
Bagging	II	Not applicable	176721	2755074	3403575	+	IL *vs.* IL
	II	4147051	4800868	10414073	11180836	+	IL *vs.* IL
	IV	3067374	3920366	3920366	4991858	+	IL *vs.* IL

QTL limits are shown by the locations of the flanking markers with N2 genotype and the adjacent markers with a CB4856 genotype. QTL marked as Single IL were detected in comparisons between ILs and N2, those marked IL *vs.* IL were detected in comparisons between ILs. Only ILs on chromosome II and IV were tested. QTL, quantitative trait loci.

There was no overall correlation between the traits assayed, showing that multiple independent functional allelic differences exist between N2 and CB4856 (on chromosomes II and IV). These analyses do however indicate that QTL affecting bagging, lifetime fecundity and lifespan can be identified in regions associated with the production of late stage progeny (Figure 6). These data also provide direct evidence for epistatic interactions affecting both lifespan and fecundity on chromosome *II*, with the IL *vs.* IL analyses of ewlR021-23 indicating an epistatic interaction between the CB4856 region in ewlR021 and the region in ewlR023 (Figure 6 and Table 2).

## Discussion

Within the *Caenorhabditis* species, there is a continuum between distinct, reproductively isolated, species and species where isolates are at the very earliest stages of speciation (Baird and Stonesifer 2012; Kozlowska *et al.* 2012; Gimond *et al.* 2013). The polymorphisms that result in outbreeding depression and hybrid breakdown within species underlie developmental transitions that can ultimately lead to speciation. Our analyses of ILs and RILs derived from the isolates CB4856 and N2 indicate that many of these lines phenocopy mild *egl* mutations, laying progeny at an advanced stage of development (Figure 1). Genetic analyses of these data revealed multiple QTL affecting egg-laying (Figure 2 and Table 1). These data indicate that the stage at which an egg is deposited is a polygenic trait. However, it is not clear from this analysis if this is a consequence of the additive action of positive and negative allele(s) from CB4856, of epistatic interactions between loci, or a combination of both. The observation that all of the wild isolates lay very early stage eggs (Figure 4) and that the QTL are associated with increased bagging does however argue that laying late stage eggs is deleterious and therefore that selection will be acting to minimize this.

The other phenotypes linked to these *egl* effects involve fitness traits (Table 2). The clearest association is with bagging, with ILs underlying the QTL on both chromosomes showing increased bagging (Figure 6 and Table 2). This association between laying late stage progeny and an increased rate of bagging is unsurprising given that this is a common phenotype in *egl* mutants (Trent *et al.* 1983). The patterns of bagging observed on both chromosome II and IV indicates that these do not represent simple interactions between two loci. For example, comparison of ILs ewIR21-23 (Figure 6) suggest the presence of interactions with other loci on the same chromosome (*e.g.*, between alleles present in ewLR21 and those in ewlR23). As this trait is, like production of late-stage embryo trait, based on the proportion of the population showing the trait, it is not possible to use these comparisons to distinguish between QTL acting additively and those that are a function of epistatic interactions. This is not the case for the lifespan and fecundity QTL that we detect in the two incompatibility regions (Table 2), as positive effect QTL would be detected in comparisons between ILs and N2. Here, both regions support the interpretation of the QTL as epistatic interactions. For instance, comparisons between ILs on chromosome II define two positive effect QTL for both fecundity and lifespan (Table 2), but the introgressions in this region are not consistent with this, as it would imply two positive effect QTL in ewlR22 and one each in ewlR21 and 23 (Figure 6). Because ewlR21 and 23 are not different to N2, a more parsimonious explanation would be that the increased lifespan and fecundity seen in ewlR22 is a consequence of an interaction between CB4856 alleles that are separated in ewlR21 and 23. In this context, it noteworthy that ewlR21 has a slightly reduced lifespan in this assay and has been previously shown, using these ILs, to contain a CB4856 allele that reduces lifespan (Doroszuk *et al.* 2009). A similar case for a complex interaction can be made for the lifespan QTL identified on chromosome IV (Table 2), a QTL also found by Doroszuk *et al.* (2009). Given that fecundity QTL are detected at both ends of chromosome IV (Figure 6 and Table 2), it is not clear if a model of additive QTL is more consistent with these data than one reliant on epistatic interactions.

Given the detrimental effects of the QTL we have detected, it is likely that they would represent weak postzygotic barriers. Conceptually, the effects we have detected can be viewed in a number of differing ways. They could be the consequence of transgressive segregation, although in this case this is unlikely as the trait mapped is essentially synthetic and not seen in either parent or in other wild isolates. Alternatively, the trait could be the result of a disruption of canalization and represent the exposure of cryptic genetic variation. In general, canalization acts to limit trait sensitivity to changes in the environment and/or the genetic background (Waddington 1942; Schmalhausen 1949; Lerner 1954). Within species, such incompatibilities will appear similar to cryptic variation, a situation where genetic or environmental perturbation is required to reveal otherwise hidden genetic variation (Gibson and Dworkin 2004; Li *et al.* 2006; Masel and Siegal 2009; Snoek *et al.* 2012; Paaby and Rockman 2014). Here, the origin of cryptic variation may represent the evolution of epistatic correction of deleterious effects of a particular mutation (that may or may not also produce adaptive changes). Such changes would be analogous to the local compensatory mutations that occur both between and within species to correct structural changes in proteins (Long *et al.* 2013)

The life history of *C. elegans* may facilitate the build-up of such deleterious mutations. For example, fixation within a line of adaptive mutations that have pleiotropic deleterious effects, or mildly deleterious mutations (as aided by the extensive selfing and the bottlenecking resulting from the *C. elegans* life-history) would allow the subsequent selection for compensatory mutations. As compensatory mutations appear commonly in *C. elegans*, as shown by experiments that have reimposed selection on mutation accumulation lines (Estes and Lynch 2003; Denver *et al.* 2010; Estes *et al.* 2011), this could result in a negative interaction between the compensatory mutation and the original allele. This would produce a situation where local adaptation (first mutation advantageous) or cryptic genetic variation (first mutation deleterious and now associated with a compensatory mutation) would produce, at least, a pair of coadapted genes. In making the RILs and the ILs the links between coadapted genes might be broken up and cryptic genetic variation that only exists to correct otherwise deleterious polymorphisms is revealed. It is clear that there is significant genotypic and phenotypic variation between *C. elegans* wild isolates (Hodgkin and Doniach 1997; Viney *et al.* 2003; Barrière and Félix 2005; Barrière and Félix 2007; Harvey *et al.* 2008, 2009; Maydan *et al.* 2010; Andersen *et al.* 2012; Green *et al.* 2013; Thompson *et al.* 2013; Volkers *et al.* 2013; Snoek *et al.* 2014b). Large-scale analysis of *C. elegans* isolates reveals little grouping by isolation environment or by country of origin on a global scale (Andersen *et al.* 2012), although there is evidence at smaller scales that suggests local adaptation (Volkers *et al.* 2013). Hence, there is much potential for local adaptation to produce the kinds of interactions proposed here.

The mapping resolution of the QTL identified here precludes a detailed search for candidate genes. However, comparison of the locations of the QTL identified here to the results of expression QTL (eQTL) studies of lines produced from crosses between N2 and CB4856 (Li *et al.* 2006; Rockman *et al.* 2010; Viñuela *et al.* 2010, 2012; Snoek *et al.* 2013; Van Der Velde *et al.* 2014) suggest that a number of the genome hotspots for trans acting eQTL do co-localize with incompatibility QTL. This is particularly the case with the incompatibility QTL on the top of chromosome IV (Figure 2), where a very strong eQTL hotspot has been identified under a range of conditions (Rockman *et al.* 2010; Viñuela *et al.* 2010, 2012). This part of chromosome IV also contains multiple QTL affecting dauer larvae development in growing populations (Green *et al.* 2013) and a large number of separate QTL affecting olfactory preference between *Serratia marcescens*, a bacterium pathogenic to *C. elegans*, and *E. coli* (Glater *et al.* 2014). The large number of phenotypes now known to be linked this region and the observed complexity of their regulation, as implied by the number of separable QTL in the region (Green *et al.* 2013; Glater *et al.* 2014) (Table 1 and Figure 5), mean that determining how these variants are related will be interesting for their potential role in speciation. More generally, given the extensive lab adaptation observed in the N2 isolate (McGrath *et al.* 2009; Weber *et al.* 2010; Duveau and Félix 2012) it would be informative to investigate the role of these changes in the incompatibilities observed here as such alleles are known to be of recent origin. This would therefore demonstrate that short periods of strong selection can rapidly produce incompatibilities.

To date, the mechanisms that isolate four *Caenorhabditis* species, *C. elegans*, *C. briggsae*, *C. remanei*, and *C. sp*. strain CB5161, now named *C. brenneri* (Sudhaus and Kiontke 2007), have been described (Baird *et al.* 1992). Work on more recently isolated *Caenorhabditis* species, which can form viable, and in some cases fertile, hybrids, has also started to address the genetic bases of speciation in this group (Baird and Stonesifer 2012; Kozlowska *et al.* 2012; Gimond *et al.* 2013). Because outbreeding depression is also observed in the other predominantly self-fertilizing *Caenorhabditis* species (Ross *et al.* 2011; Baird and Stonesifer 2012; Kozlowska *et al.* 2012; Gimond *et al.* 2013) it is likely that BDM incompatibilities will also be detectable within these species. Over the longer term, the identification of the causative loci for the QTL identified here would allow comparison with the changes that produce more extreme reproductive isolation and the alleles involved in the very early stages of speciation that have been detected in other *Caenorhabditis* species (Dey *et al.* 2012; Kozlowska *et al.* 2012). This suggests that the *Caenorhabditis* species have the potential to be hugely informative about the genetics of speciation and more generally about the role of epistatic interactions in the control of complex traits.

## Supplementary Material

Supporting Information
