# Supplementary material for: Widespread Genomic Incompatibilities in Caenorhabditis elegans
Source: G3 (Bethesda). 2014 Aug 15;4(10):1813–23. doi: 10.1534/g3.114.013151 (PMC4199689; doi:10.1534/g3.114.013151)
Supplement: Supporting Information [file supp_4_10_1813__index.html]

Widespread Genomic Incompatibilities in Caenorhabditis elegans — Supporting Information 

# Widespread Genomic Incompatibilities in *Caenorhabditis elegans*

## Supporting Information for Snoek *et al.*, 2014

**Files in this Data Supplement:**

- Supporting Information - Figures S1-S4 and Table S1 (PDF, 1.6 MB)
- Figure S1 - Progeny stage distribution across the RILs and ILs (PDF, 301 KB)
- Figure S2 - MQM analysis of the RILs (PDF, 218 KB)
- Figure S3 - Comparison between ILs in the genomewide screen (PDF, 908 KB)
- Figure S4 - Egg-stages in single N2 dishes used in this paper (PDF, 167 KB)
- Table S1 - IL vs IL Egg-stages (PDF, 158 KB)
