# Supplementary material for: Widespread Genomic Incompatibilities in Caenorhabditis elegans
Source: G3 (Bethesda). 2014 Aug 15;4(10):1813–23. doi: 10.1534/g3.114.013151 (PMC4199689; doi:10.1534/g3.114.013151)
Supplement: Supporting Information [file supp_g3.114.013151_013151SI.pdf]

G3 Genes | Genomes | Genetics

Widespread genomic incompatibilities in *Caenorhabditis elegans*

L. Basten Snoek[*,1], Helen E. Orbidans[§,1], Jana J. Stastna[§], Aafke Aartse[*], Miriam Rodriguez[*], Joost A.G. Riksen[*], Jan E. Kammenga[*,2] and Simon C. Harvey[§,2]

[*] Laboratory of Nematology, Wageningen University, 6708 PB Wageningen, The Netherlands.
[§] Biomolecular Research Group, School of Human and Life Sciences, Canterbury Christ Church University, North Holmes Road, Canterbury, CT1 1QU, UK.

[1] These authors contributed equally to this work.
[2]Corresponding authors: Laboratory of Nematology, Wageningen University, 6708 PB Wageningen, The Netherlands. Email: Jan.Kammenga@wur.nl; Biomolecular Research Group, School of Human and Life Sciences, Canterbury Christ Church University, North Holmes Road, Canterbury, CT1 1QU, UK. Email: Simon.Harvey@canterbury.ac.uk.

Data is archived in WormQTL (http://www.wormqtl.org).

DOI: 10.1534/g3.114.013151

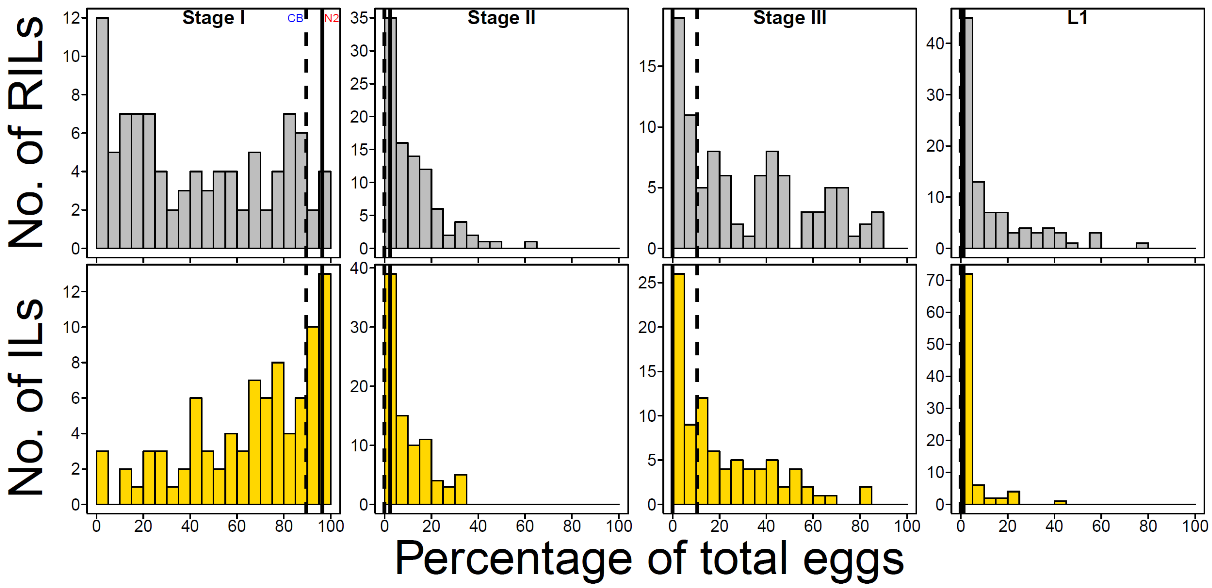

Figure S1 Progeny stage distribution across the RILs and ILs. The frequency distribution of progeny laid at a certain stage as a percentage of all progeny per genotype in the RILs (A) in grey and ILs (B) in yellow. The parental phenotypes are indicated by the vertical lines, N2 solid, CB4856 dashed. Progeny stage is indicated above each panel.

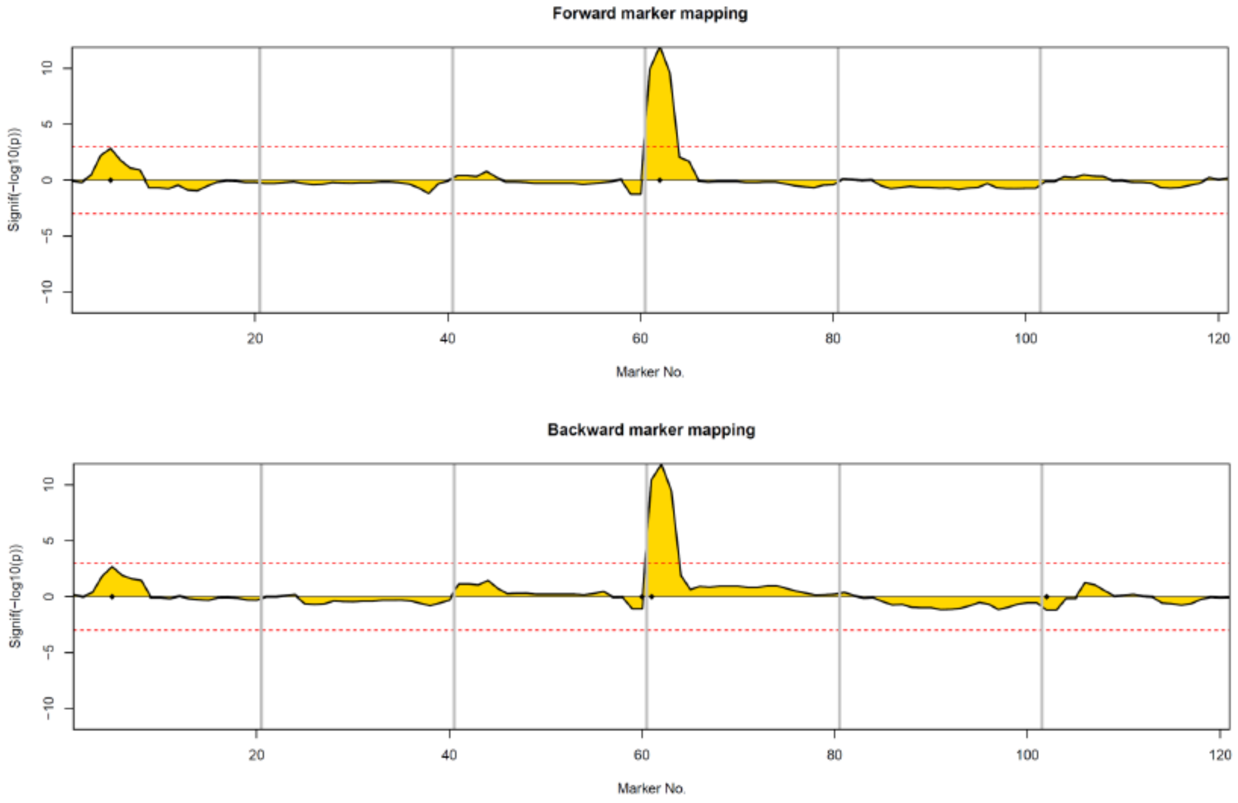

Figure S2 MQM analysis of the RILs. Percentage of stage I and II eggs per RIL was used as input for MQM QTL mapping. We used both forward mapping and backward mapping indicated in the figure titles. For forward mapping cofactor markers were added until no significant new QTL were found, for backward mapping 5 equally spaced co-factor marker were used in the starting model and each round the least significant co-factor was removed until no co-factors with p > 0.1 remained. For both forward and backward MQM mapping a window-size of 5 markers was used in calculating the final QTL profiles. We also fixed the major QTL at the top of chromosome IV to observe the QTL profiles of a N2 or CB fixed major QTL.

Figure S2 (Continued)

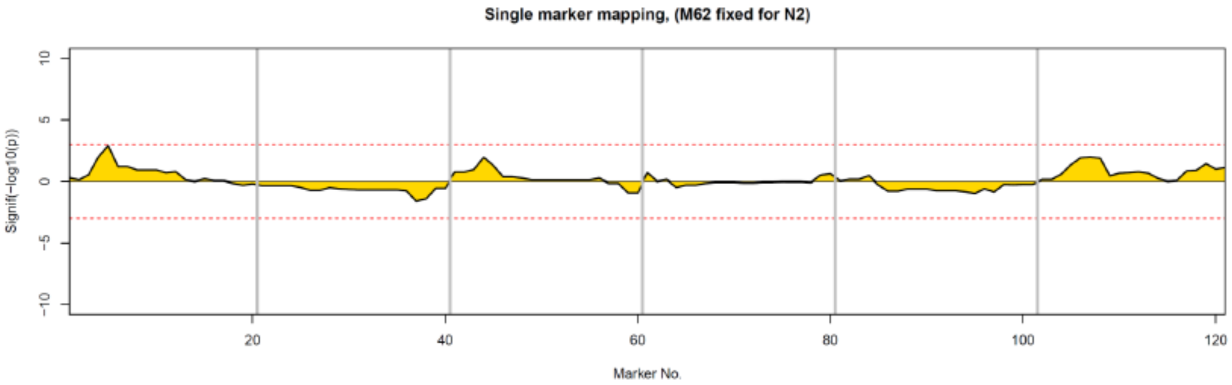

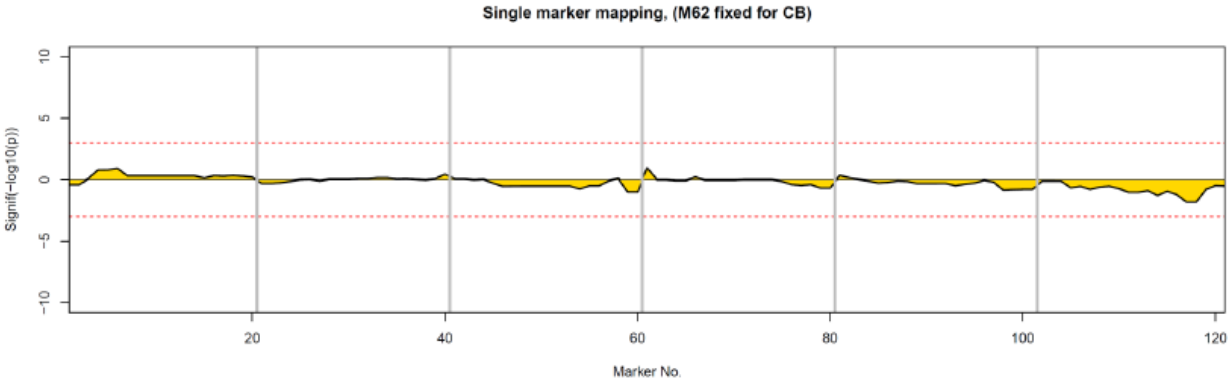

Figure S2 (Continued)

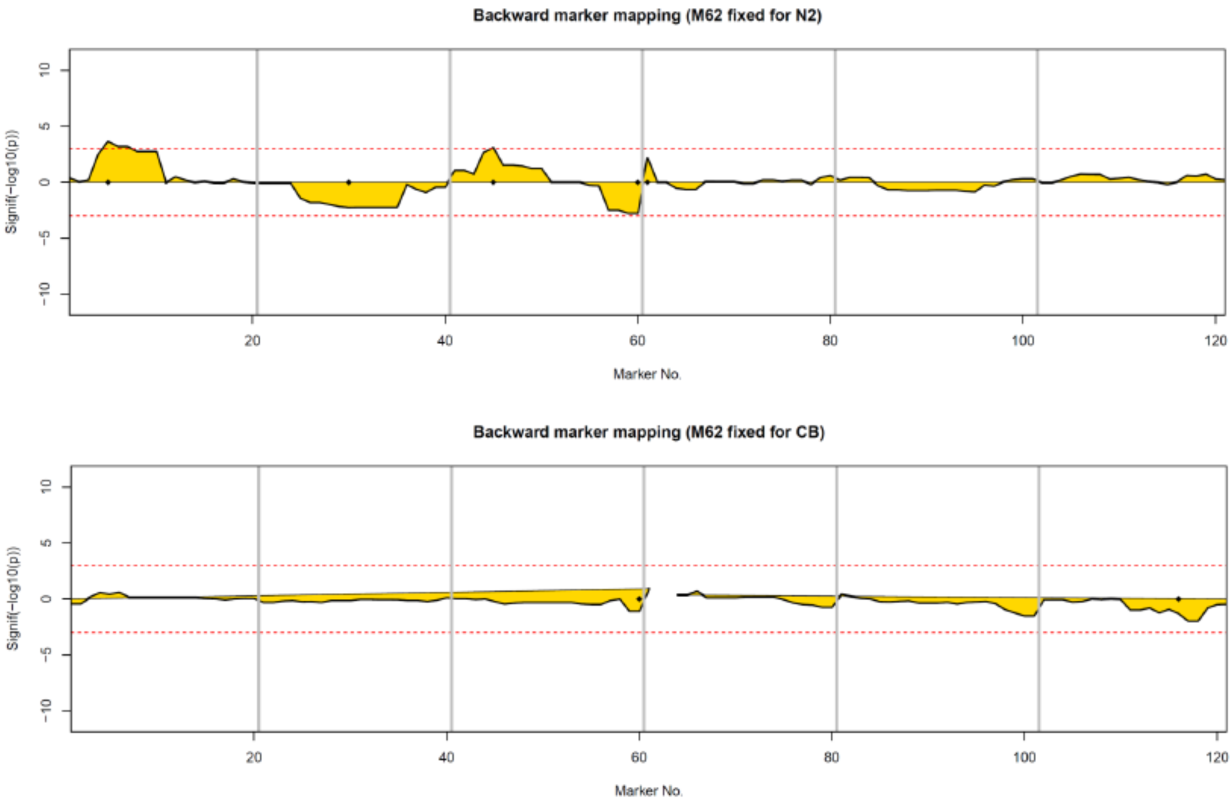

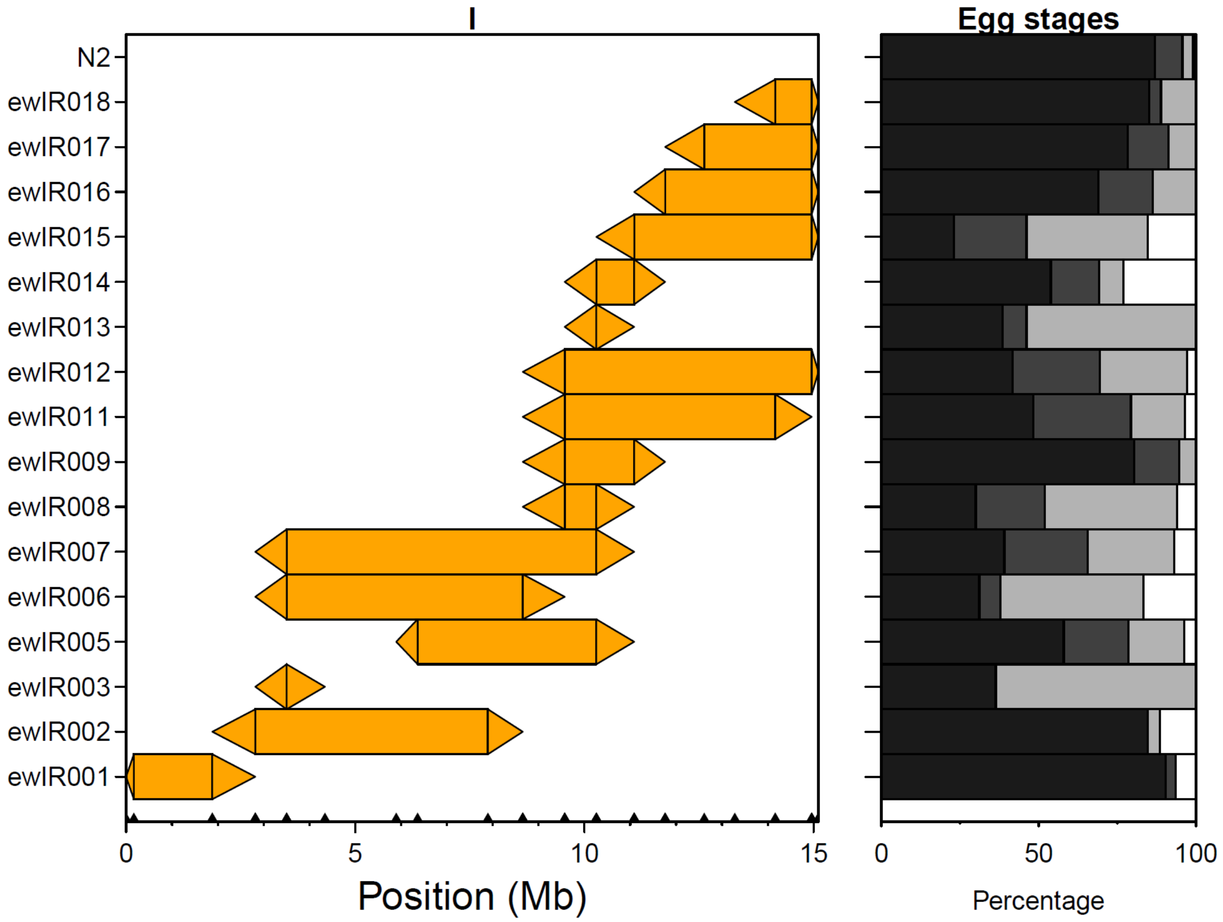

Figure S3 Comparison between ILs in the genomewide screen. The CB4856 introgression per IL is shown by the coloured rectangle. Triangles join adjacent CB4856 and N2 markers. Progeny stage distribution is shown as cumulative percentage of total progeny. From dark to light: Stage I, II, III and L1 (in white).

Figure S3 (Continued)

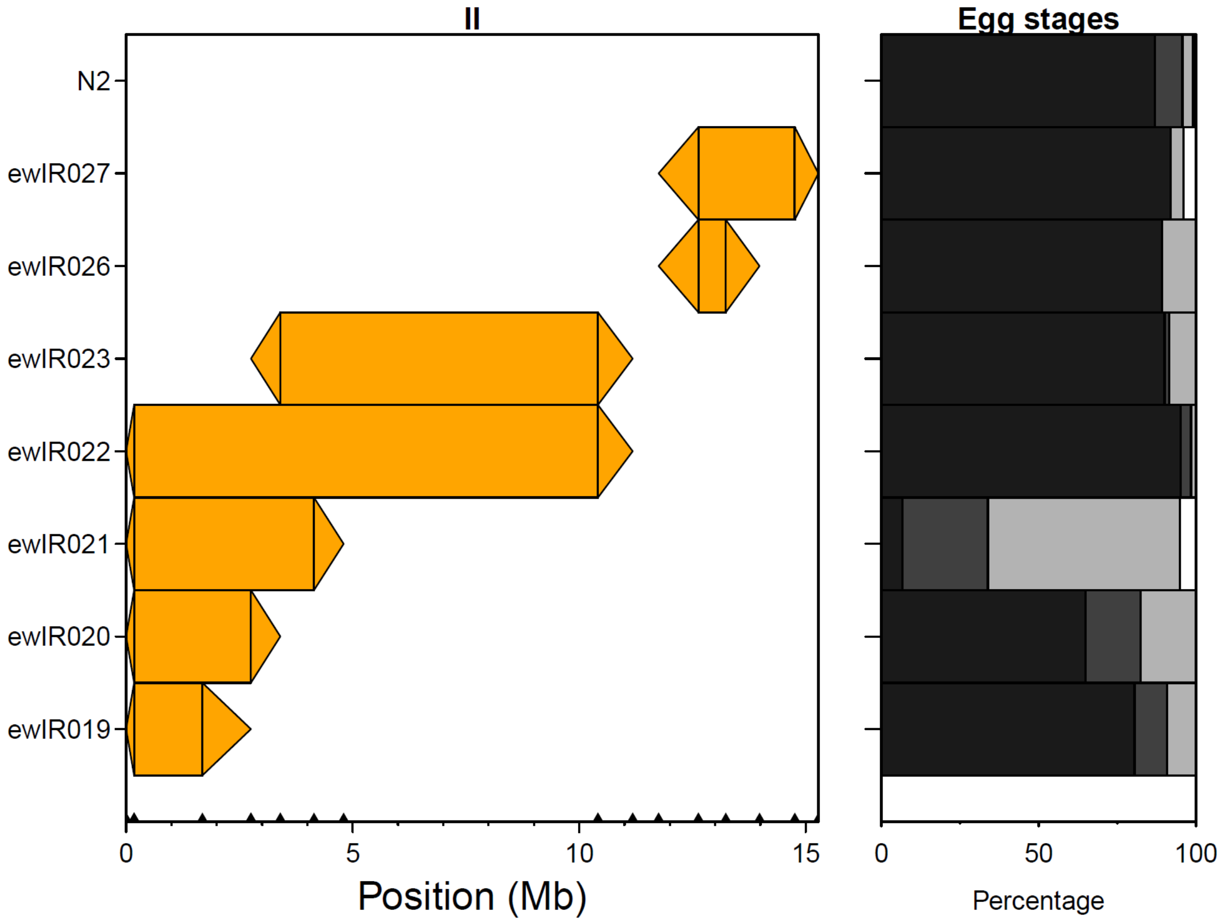

Figure S3 (Continued)

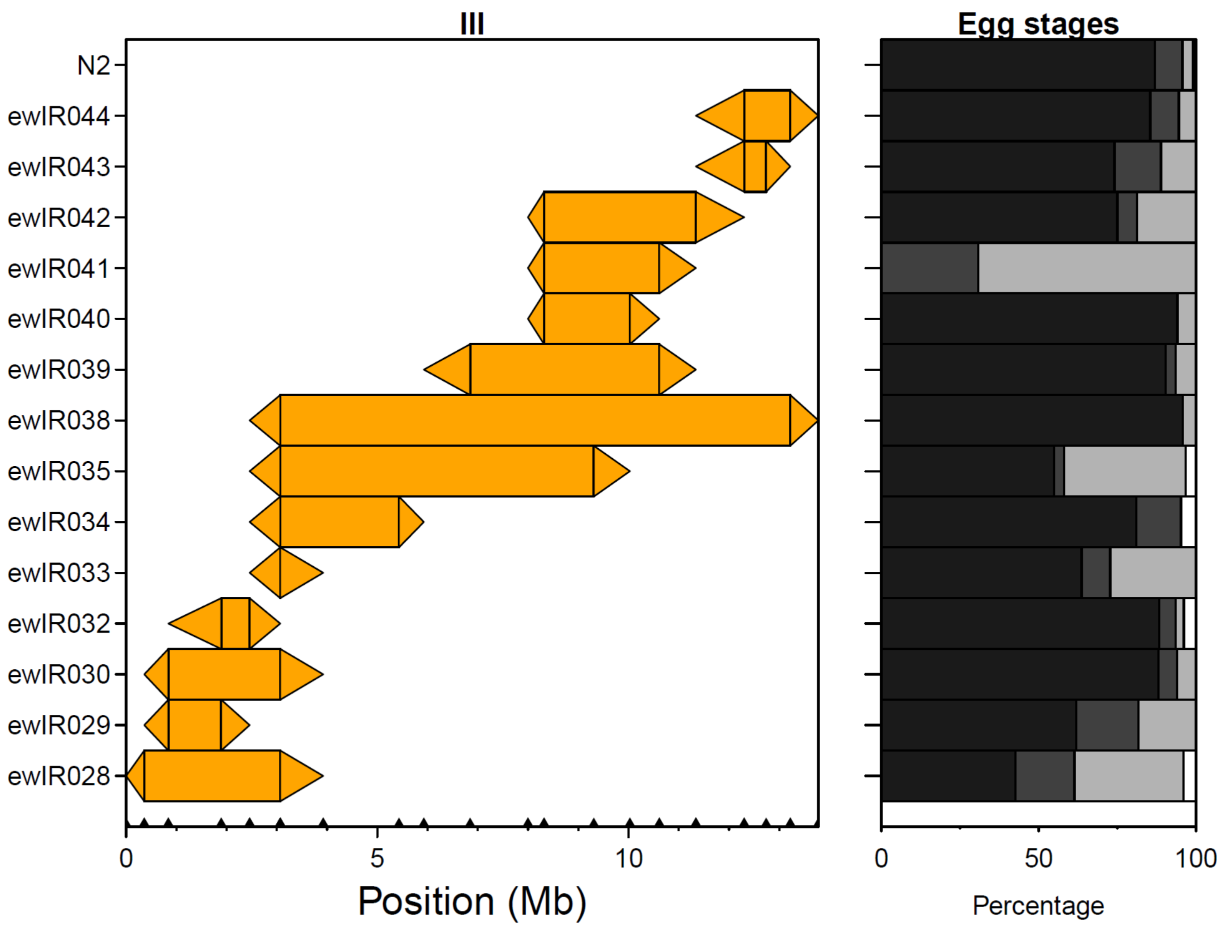

Figure S3 (Continued)

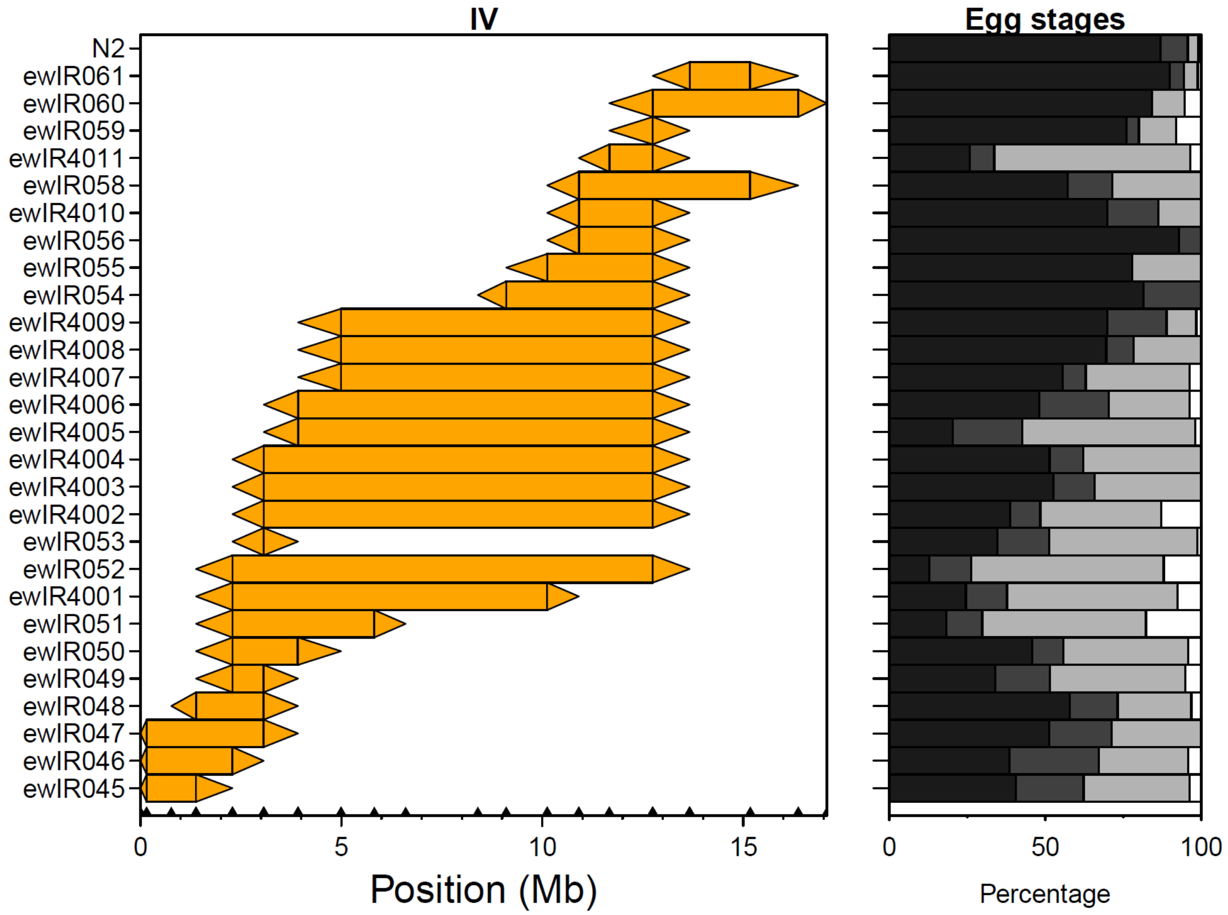

Figure S3 (Continued)

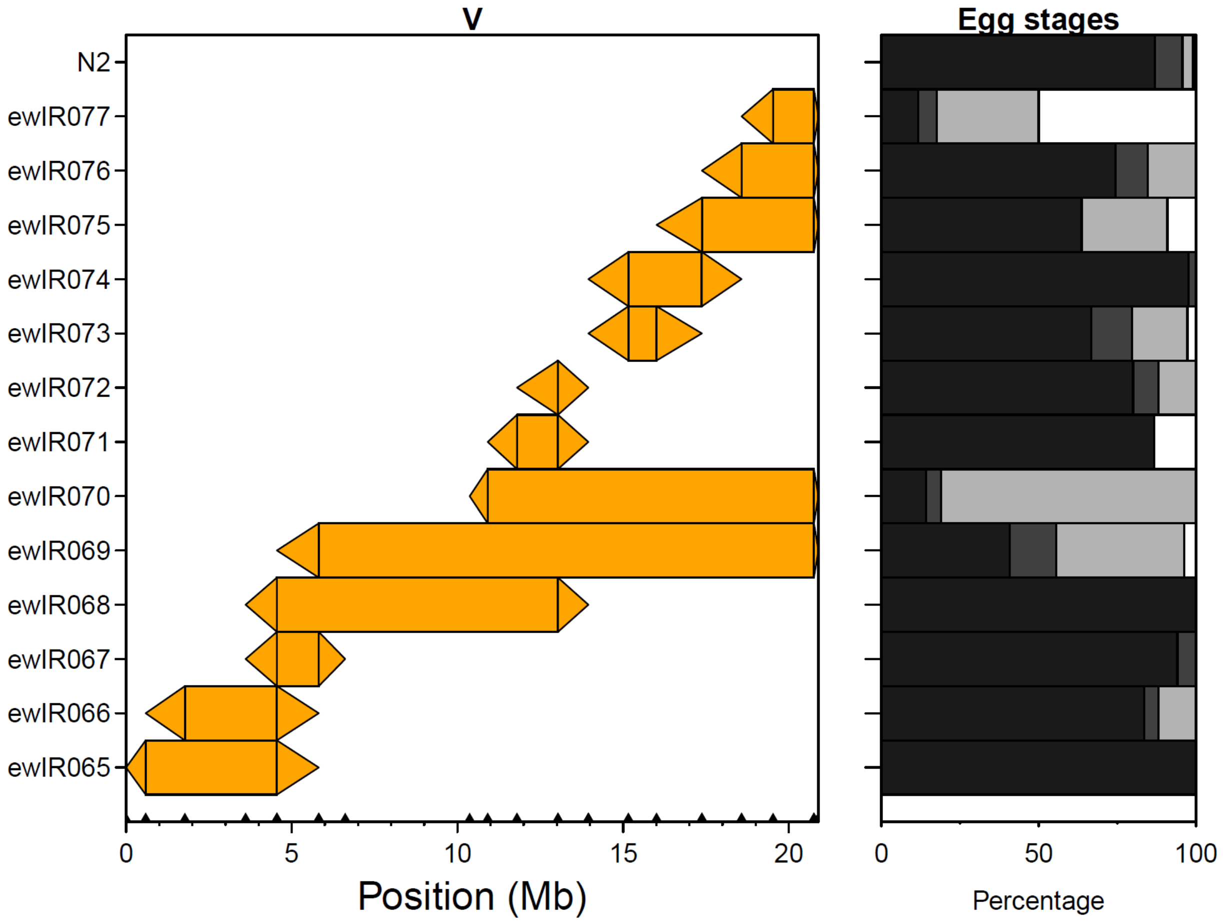

Figure S3 (Continued)

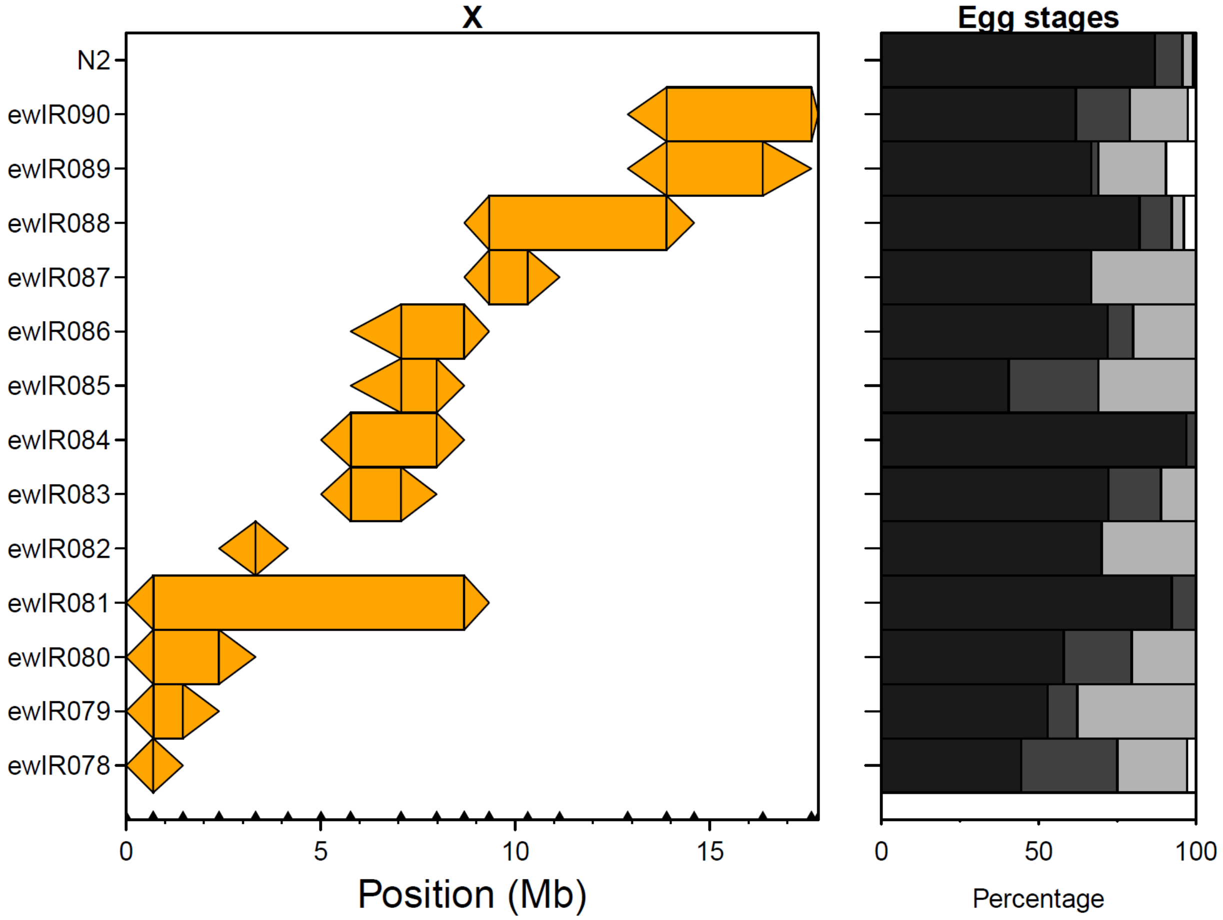

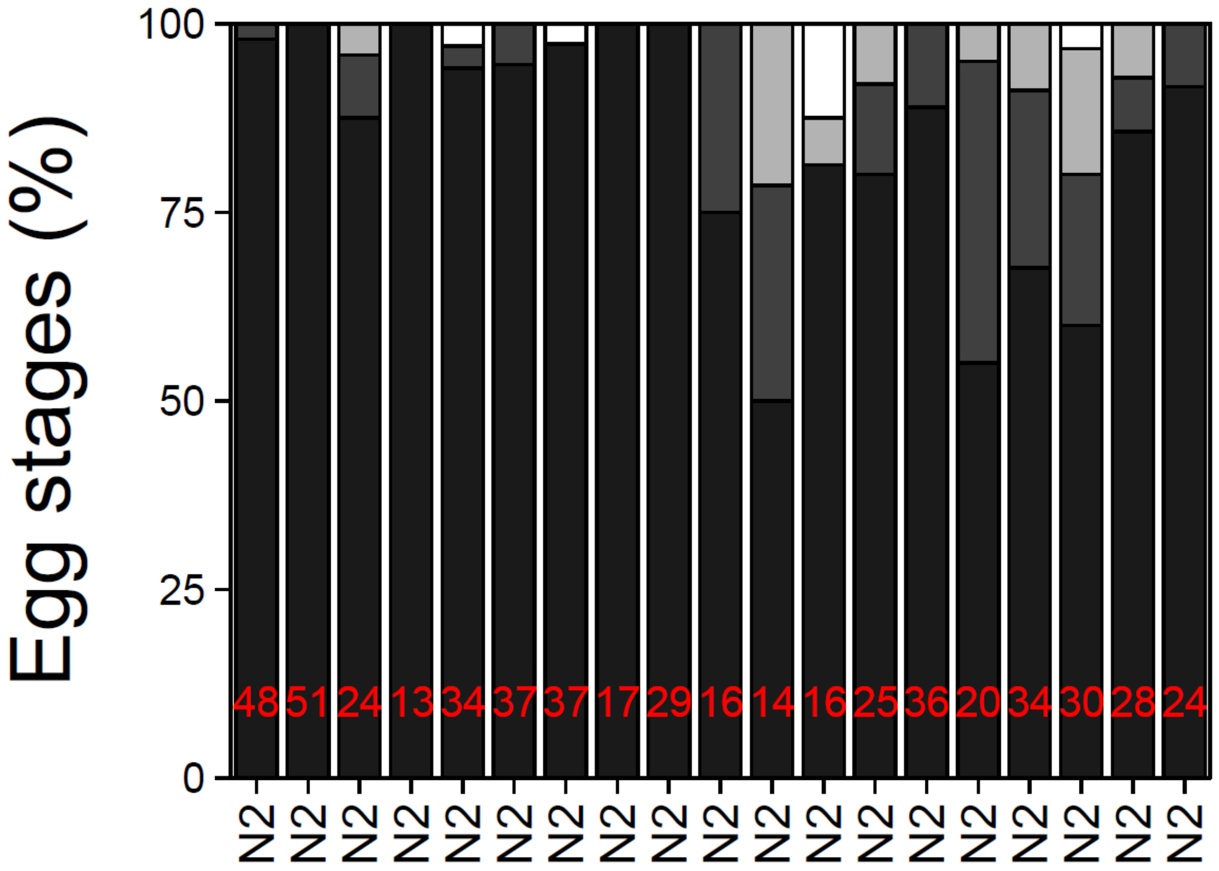

Figure S4 Egg-stages in single N2 dishes used in this paper. The number of eggs measured is indicated in red. Progeny stage distribution is shown as cumulative percentage of total progeny. From dark to light: Stage I, II, III and L1 (in white).

Table S1 IL vs IL Egg-stages. Significance scores in –log10(p)

ID	Test order	Scores (chi-square test)	N2L	CBL	CBR	N2R	CB_late_stage
1	N2 → 1	1.76					
2	N2 → 2	3.33					
3	N2 → 3	17.51	2818974	3502476	3502476	4338254	+
3	3 → 6	5.17					
3	6 → 7	4.01					
4	N2 → 13	13.13	9569913	10259909	10259909	11085295	+
4	13 → 8	2.82					
4	8 → 5	3.62					
4	5 → 7	1.23					
5	N2 → 6	15.88	2818974	3502476	8654361	9569913	+
6	N2 → 13	13.13	9569913	10259909	10259909	11085295	+
6	13 → 14	12.83	11085295	11085295	11085295	11760179	-
7	N2 → 8	14.34	8654361	9569913	10259909	11085295	+
7	8 → 9	12.02	11085295	11085295	11085295	11760179	-
7	9 → 11	4.56					
7	11 → 12	0.44					
8	N2 → 18	1.18					
8	18 → 17	1.26					
8	17 → 16	0.5					
8	16 → 15	10.26	10259909	11085295	11085295	11760179	+
8	15 → 12	3.0					
9	N2 → 19	0.58					
9	19 → 20	1.33					
9	20 → 21	16.16	2755074	3403575	4147051	4800868	+
9	21 → 22	33.06	4147051	4800868	10414073	11180836	-
10	N2 → 23	1.45					
10	23 → 22	1.21					
11	N2 → 26	2.46					
11	26 → 27	1.54					
12	N2 → 33	4.43					
12	33 → 30	3.94					

12	30 → 28	9.34					
13	N2 → 29	3.68					
13	29 → 30	3.92					
14	N2 → 32	0.38					
15	N2 → 33	4.43					
15	33 → 34	6.97					
15	34 → 35	10.31	5431252	5925983	9308858	10027496	-
15	35 → 38	9.13					
16	N2 → 41	33.67	7998164	8318553	10613119	11341120	+
16	41 → 39	35.77	5925983	6847169	7998164	8318553	-
17	N2 → 40	1.88					
17	40 → 41	38.72	10027496	10613119	10613119	11341120	+
17	41 → 42	26.10	10613119	11341120	11341120	12301725	-
18	N2 → 43	1.26					
18	43 → 44	0.89					
19	N2 → 44	0.16					
20	44 → 38	2.14					
21	N2 → 45	9.89	0	151889	1381409	2288742	+
21	45 → 46	0.16					
21	46 → 47	1.26					
22	N2 → 53	12.90	2288742	3067374	3067374	3920366	+
22	53 → 49	0.32					
22	49 → 48	2.25					
22	48 → 47	0.71					
2	N2 → 49	12.88	1381409	2288742	3067374	3920366	+

3							
23	49 → 50	0.58					
23	50 → 51	4.17					
23	51 → 4001	0.80					
23	4001 → 52	0.80					
24	N2 → 53	12.90	2288742	3067374	3067374	3920366	+
24	53 → 4002,4003,4004	2.23,1.25,1.12					
25	N2 → 59	2.32					
25	59 → 4011	12.72	10909560	11668242	11668242	12748880	+
25	4011 → 4010,56	11.89,21.71	10122930	10909560	10909560	11668242	-
25	4010,56 → 55	6.67,4.10					
25	55 → 54	8.87					
25	54 → 4009,4008,4007	2.13,5.69,9.40					
25	4009,4008,4007 → 4006,4005	4005 vs 4009 12.22					
25	4006,4005 → 4004,4003,4002	NS					
25	4004,4003,4002 → 52	NS					
26	N2 → 56,4010	0.71,2.00					
26	56,4010 → 58	8.57,1.45					
27	N2 → 59	2.32					
27	59 → 60	0.78					
28	N2 → 61	0.19					
29	N2 → 66	2.55					
29	66 → 65	3.88					

30	N2 → 67	0.80					
30	67 → 68	1.39					
31	N2 → 72	0.97					
31	72 → 71	6.62					
31	71 → 68	3.33					
32	N2 →77	26.95	18574593	19525561	20758352	20893784	+
32	77 → 76	21.39	17377158	18574593	18574593	19525561	-
32	76 → 75	4.50					
32	75 → 70	14.74	10368660	10912994	16008404	17377158	+
32	70 → 69	6.89					
33	N2 → 73	2.54					
33	73 → 74	6.60					
34	N2 → 78	8.08					
34	78 → 79	3.50					
34	79 → 80	2.20					
34	80 → 81	7.58					
35	N2 → 83	1.50					
35	83 → 84	5.25					
36	N2 → 85	10.19	5770179	7067019	7982354	8691677	+
36	85 → 84	16.25	5010049	5770179	5770179	7067019	-
37	N2 → 87	7.31					
37	87 → 88	7.78					
38	N2 → 89	5.13					
38	89 → 90	2.83					

IL vs IL Size

ID	Test order	Scores (t-test)	N2L	CBL	CBR	N2R	CB_Size
9	N2 → 19	0.27					
9	19 → 20	0.67					
9	20 → 21	0.29					
9	21 → 22	0.40					
10	N2 → 23	0.46					
10	23 → 22	0.01					
11	N2 → 26	0.59					
11	26 → 27	0.52					
21	N2 → 45	0.10					
21	45 → 46	0.68					
21	46 → 47	0.19					
22	N2 → 53	0.29					
22	53 → 49	0.03					
22	49 → 48	1.11					
22	48 → 47	0.31					
23	N2 → 49	0.42					
23	49 → 50	0.08					
23	50 → 51	0.26					
23	51 → 52	1.30	5819735	6599685	12748880	13667267	-
24	N2 → 53	0.29					
25	N2 → 59	1.45	11668242	12748880	12748880	13667267	+
25	59 → 56	1.08					
25	56 → 55	0.34					
25	55 → 54	1.37	8397264	9102404	9102404	10122930	+
25	54 → 52	1.01					
26	N2 → 56	0.05					
26	56 → 58	1.18					
27	N2 → 59	1.45	11668242	12748880	12748880	13667267	+
27	59 → 60	0.03					
28	N2 → 62	1.64	12748880	13667267	16371991	17084259	-

IL vs IL Lifespan

ID	Test order	Scores (t-test)	N2L	CBL	CBR	N2R	CB_Lifespan
9	N2 → 19	0.07					
9	19 → 20	0.17					
9	20 → 21	0.46					
9	21 → 22	2.69	4147051	4800868	10414073	11180836	+
10	N2 → 23	0.09					
10	23 → 22	1.75	0	176721	2755074	3403575	+

11	N2 → 26	0.96					
11	26 → 27	0.02					
21	N2 → 45	0.20					
21	45 → 46	0.13					
21	46 → 47	0.16					
22	N2 → 53	0.89					
22	53 → 49	0.50					
22	49 → 48	0.24					
22	48 → 47	0.55					
23	N2 → 49	0.24					
23	49 → 50	0.09					
23	50 → 51	1.30	3920366	4991858	5819735	6599685	-
23	51 → 52	0.65					
24	N2 → 53	0.89					
25	N2 → 59	0.38					
25	59 → 56	0.17					
25	56 → 55	0.73					
25	55 → 54	0.26					
25	54 → 52	0.77					
26	N2 → 56	0.54					
26	56 → 58	0.13					
27	N2 → 59	0.38					
27	59 → 60	0.05					
28	N2 → 62	0.54					

IL vs IL Fecundity

ID	Test order	Scores (t-test)	N2L	CBL	CBR	N2R	CB_Fecundity
9	N2 → 19	0.11					
9	19 → 20	0.23					
9	20 → 21	0.53					
9	21 → 22	3.37	4147051	4800868	10414073	11180836	+
10	N2 → 23	0.77					
10	23 → 22	2.87	0	176721	2755074	3403575	+
11	N2 → 26	0.67					
11	26 → 27	0.98					
21	N2 → 45	0.37					
21	45 → 46	0.31					
21	46 → 47	0.62					
22	N2 → 53	0.36					
22	53 → 49	0.20					
22	49 → 48	0.77					
22	48 → 47	0.02					
23	N2 → 49	0.72					
23	49 → 50	0.59					

23	50 → 51	0.84					
23	51 → 52	0.99					
24	N2 → 53	0.36					
25	N2 → 59	0.14					
25	59 → 56	0.01					
25	56 → 55	1.14					
25	55 → 54	0.18					
25	54 → 52	0.58					
26	N2 → 56	0.13					
26	56 → 58	0.95					
27	N2 → 59	0.14					
27	59 → 60	1.34	12748880	13667267	16371991	17084259	-
28	N2 → 62	2.13	12748880	13667267	16371991	17084259	-
