# Supplementary material for: Widespread Genomic Incompatibilities in Caenorhabditis elegans
Source: G3 (Bethesda). 2014 Aug 15;4(10):1813–23. doi: 10.1534/g3.114.013151 (PMC4199689; doi:10.1534/g3.114.013151)
Supplement: Supporting Information [file supp_g3.114.013151_FigureS1.pdf]

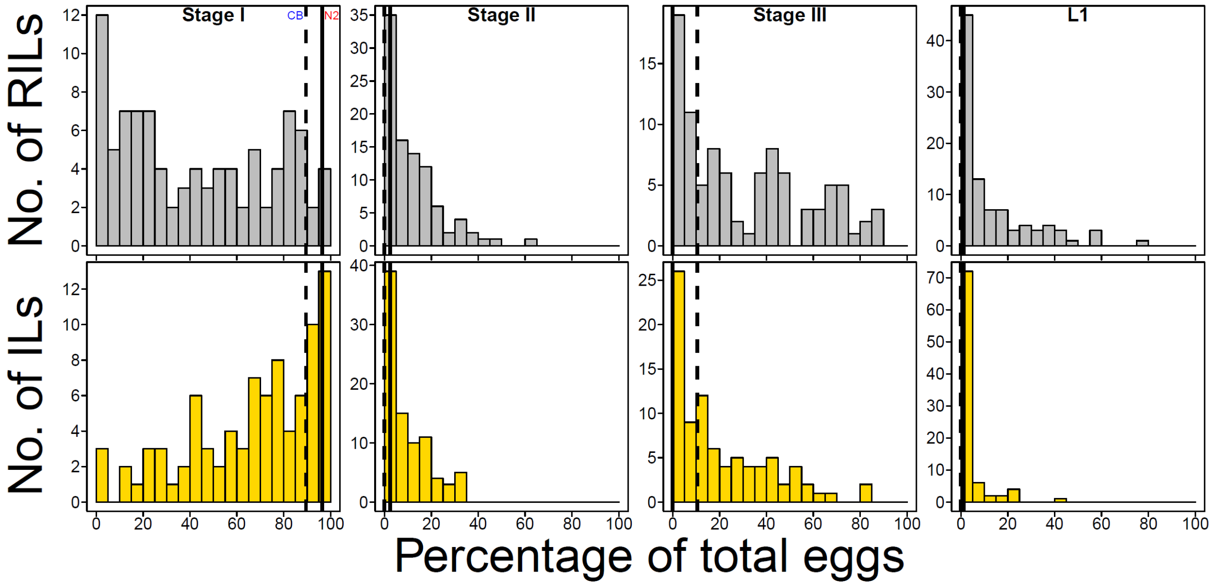

Figure S1 Progeny stage distribution across the RILs and ILs. The frequency distribution of progeny laid at a certain stage as a percentage of all progeny per genotype in the RILs (A) in grey and ILs (B) in yellow. The parental phenotypes are indicated by the vertical lines, N2 solid, CB4856 dashed. Progeny stage is indicated above each panel.
