# Supplementary material for: Widespread Genomic Incompatibilities in Caenorhabditis elegans
Source: G3 (Bethesda). 2014 Aug 15;4(10):1813–23. doi: 10.1534/g3.114.013151 (PMC4199689; doi:10.1534/g3.114.013151)

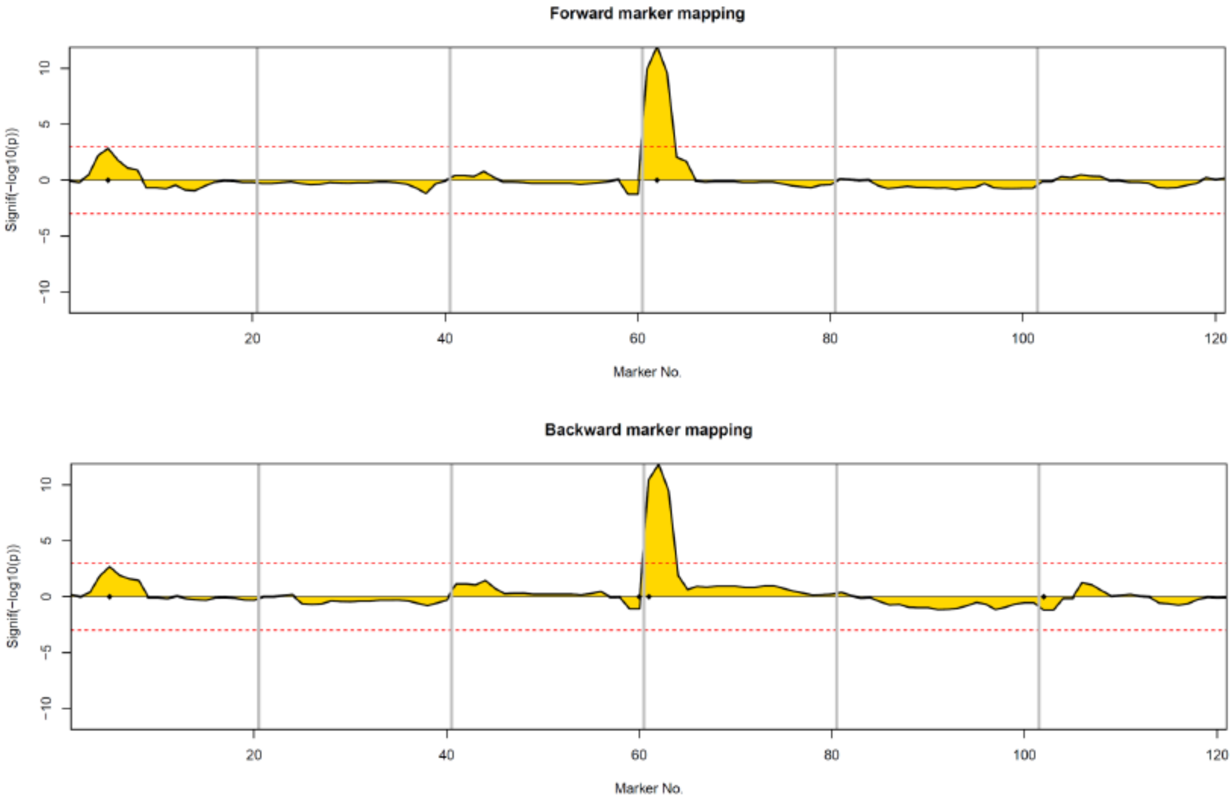

Figure S2 MQM analysis of the RILs. Percentage of stage I and II eggs per RIL was used as input for MQM QTL mapping. We used both forward mapping and backward mapping indicated in the figure titles. For forward mapping cofactor markers were added until no significant new QTL were found, for backward mapping 5 equally spaced co-factor marker were used in the starting model and each round the least significant co-factor was removed until no co-factors with p > 0.1 remained. For both forward and backward MQM mapping a window-size of 5 markers was used in calculating the final QTL profiles. We also fixed the major QTL at the top of chromosome IV to observe the QTL profiles of a N2 or CB fixed major QTL.

Figure S2 (Continued)

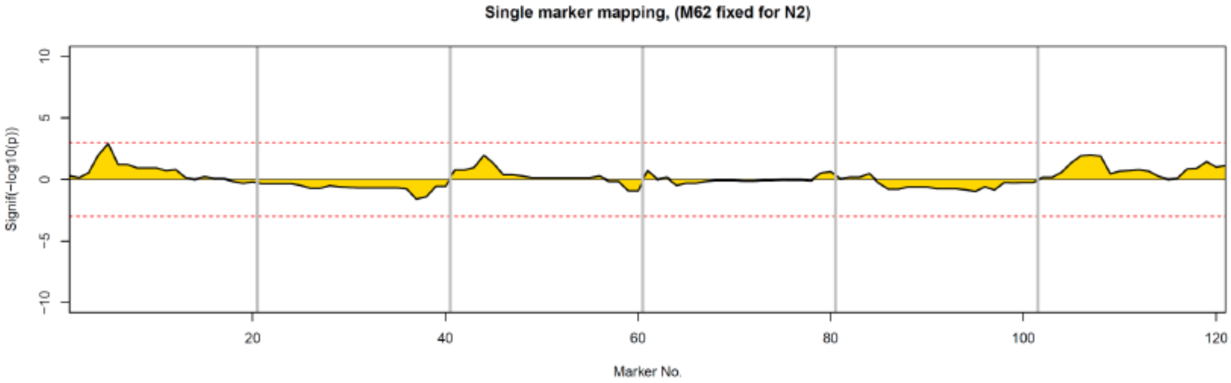

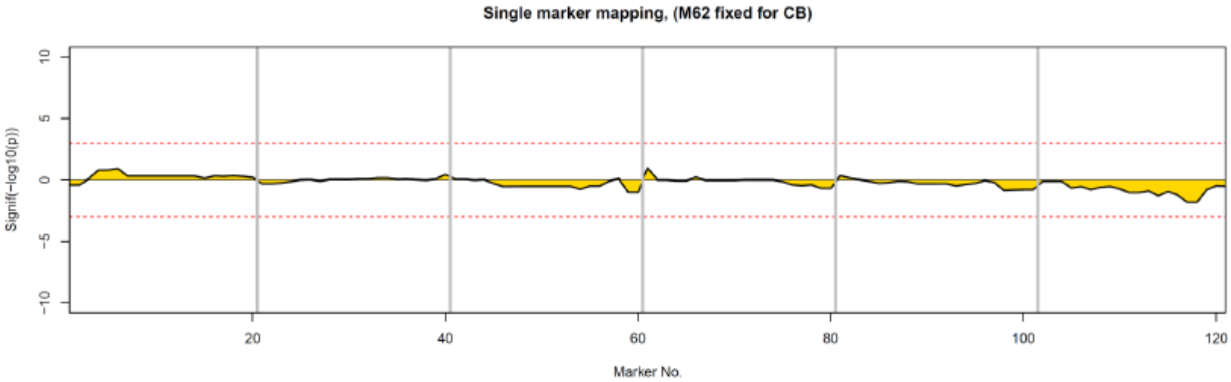

Figure S2 (Continued)

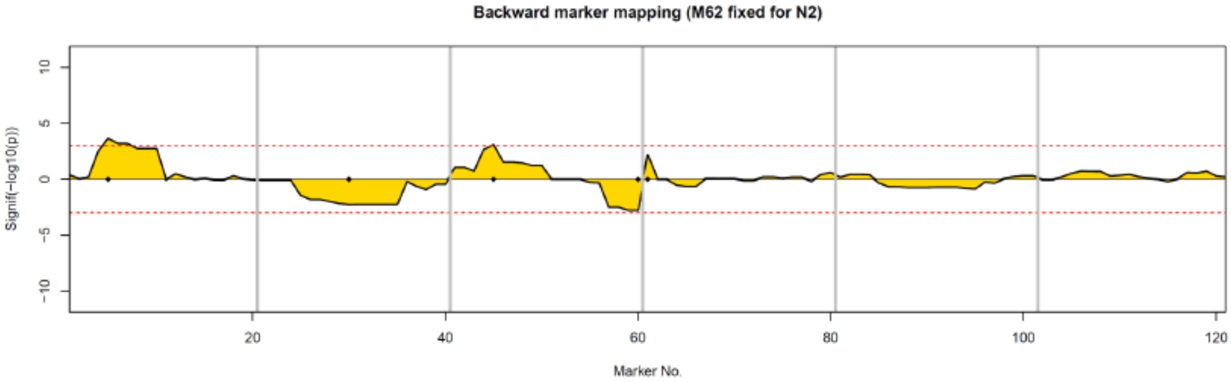

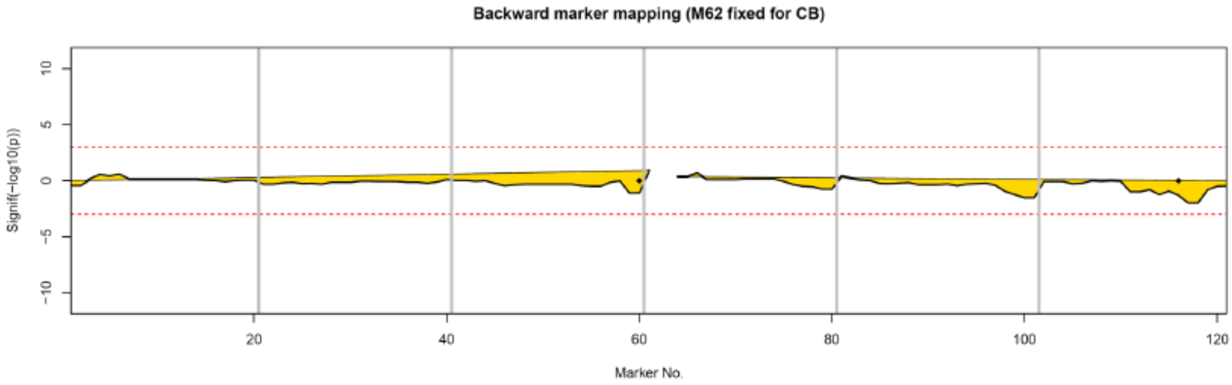

Supplement: Supporting Information [file supp_g3.114.013151_FigureS2.pdf]
