# Supplementary material for: Widespread Genomic Incompatibilities in Caenorhabditis elegans
Source: G3 (Bethesda). 2014 Aug 15;4(10):1813–23. doi: 10.1534/g3.114.013151 (PMC4199689; doi:10.1534/g3.114.013151)

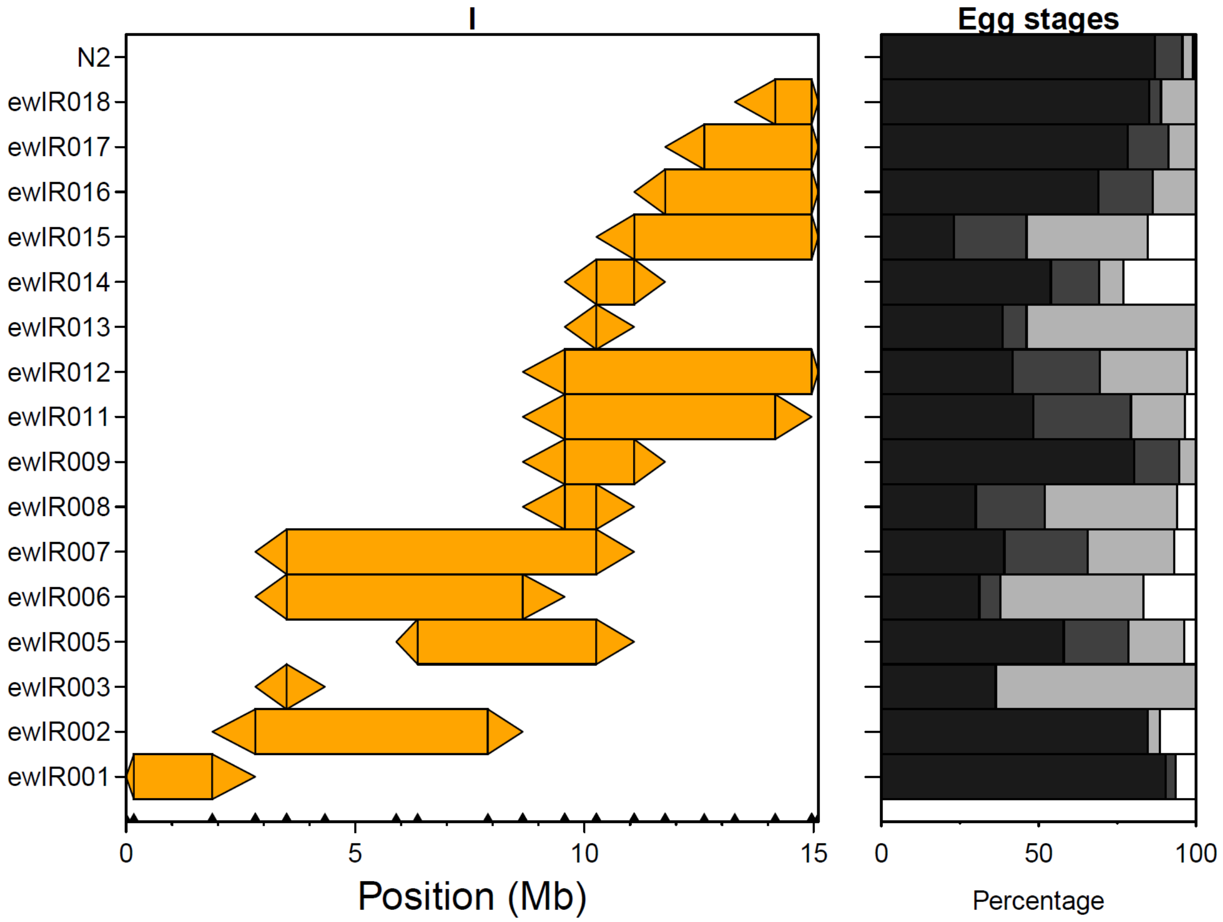

Figure S3 Comparison between ILs in the genomewide screen. The CB4856 introgression per IL is shown by the coloured rectangle. Triangles join adjacent CB4856 and N2 markers. Progeny stage distribution is shown as cumulative percentage of total progeny. From dark to light: Stage I, II, III and L1 (in white).

Figure S3 (Continued)

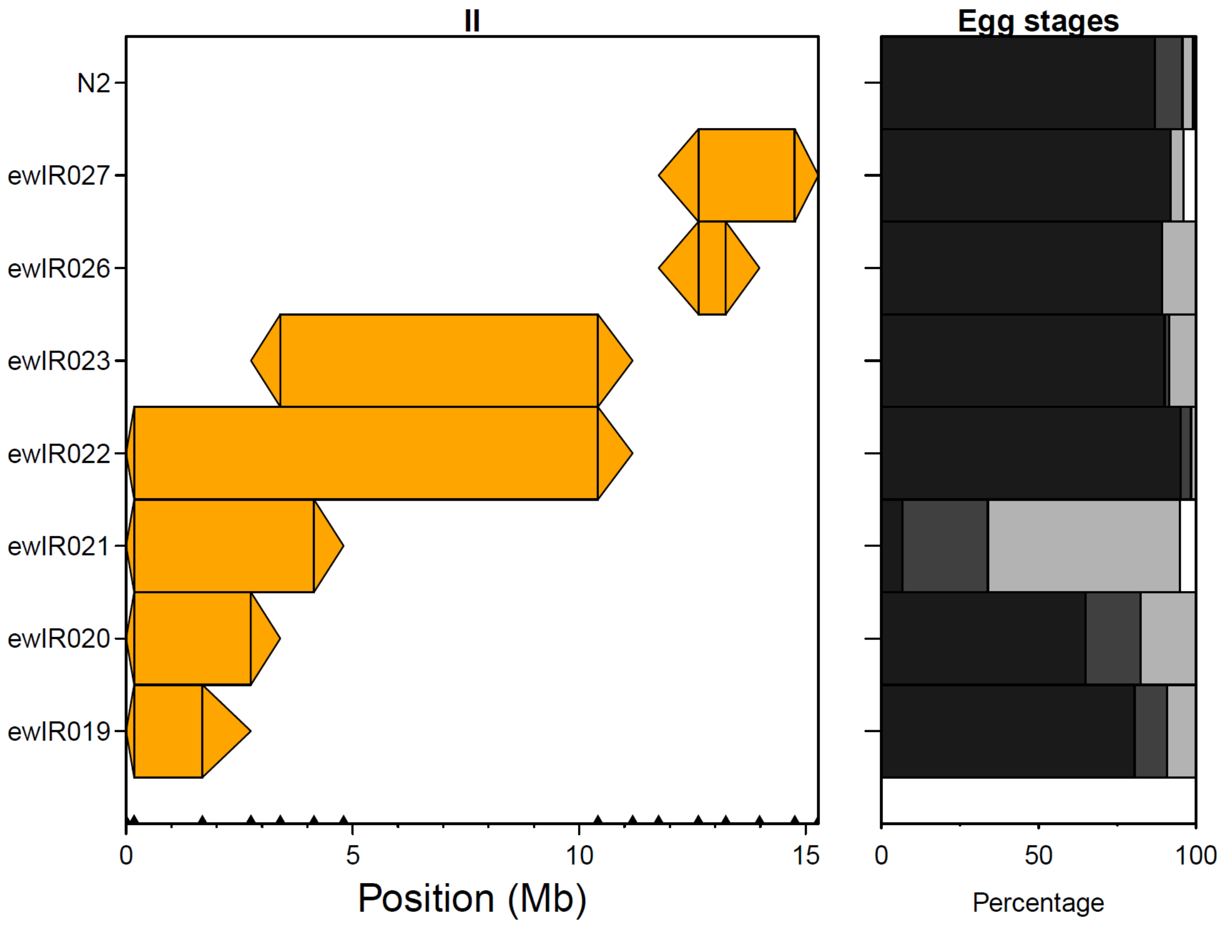

Figure S3 (Continued)

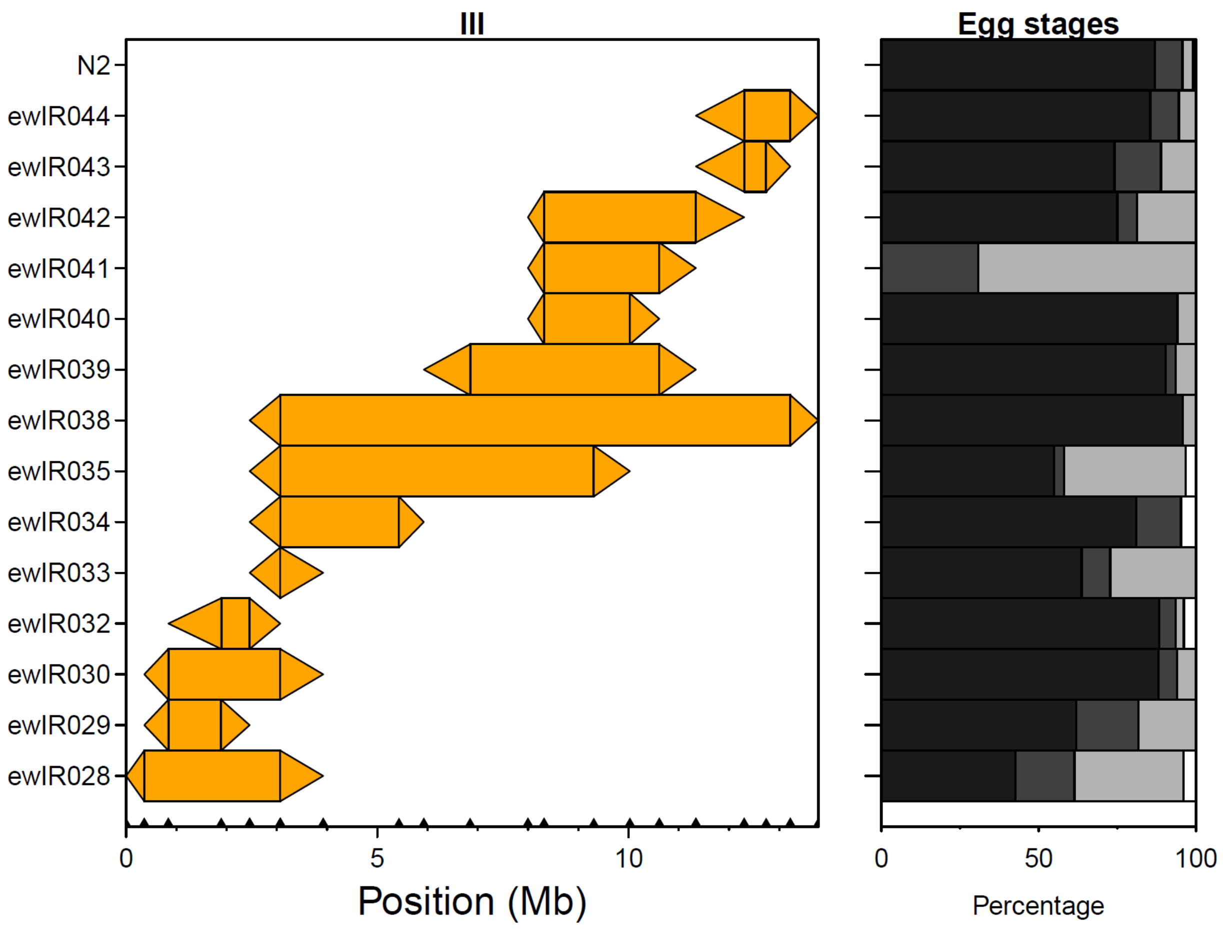

Figure S3 (Continued)

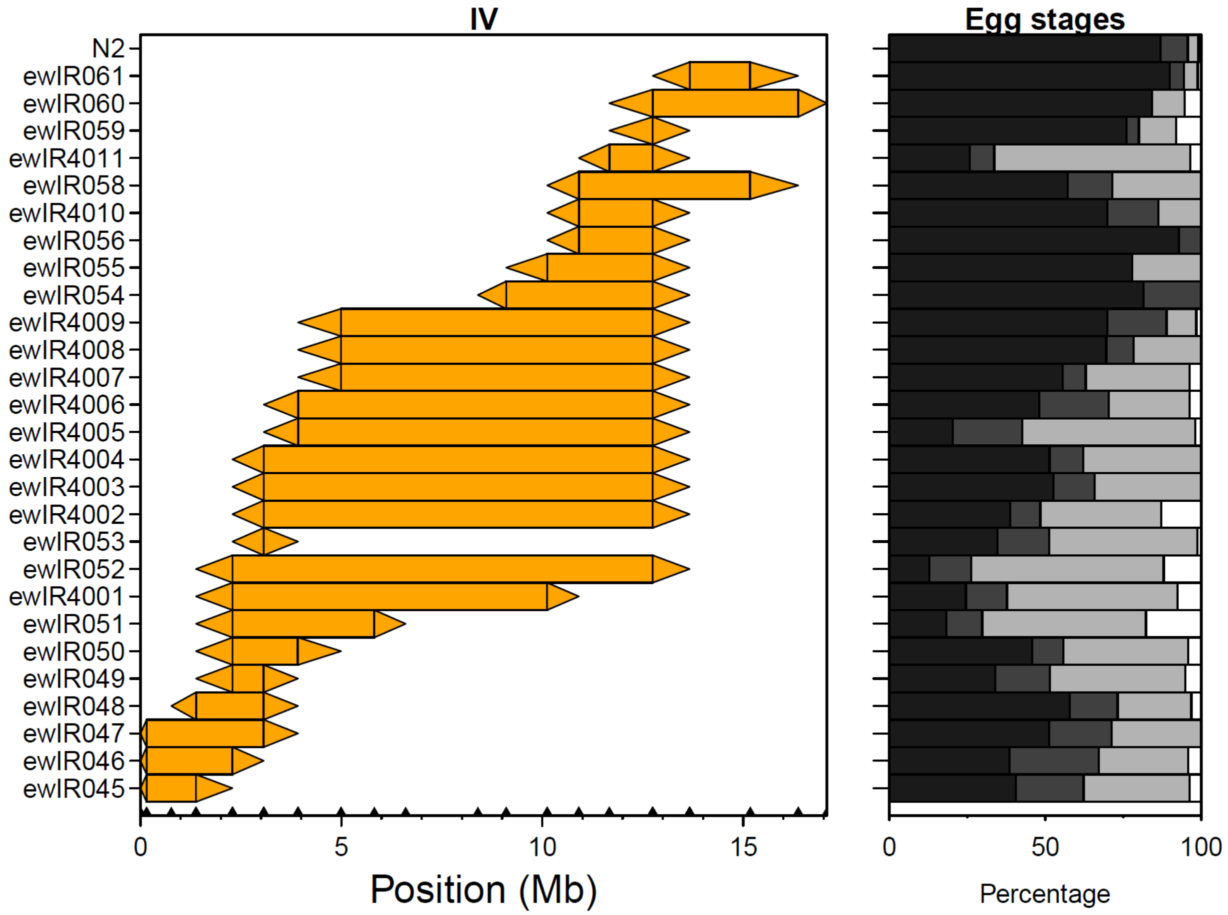

Figure S3 (Continued)

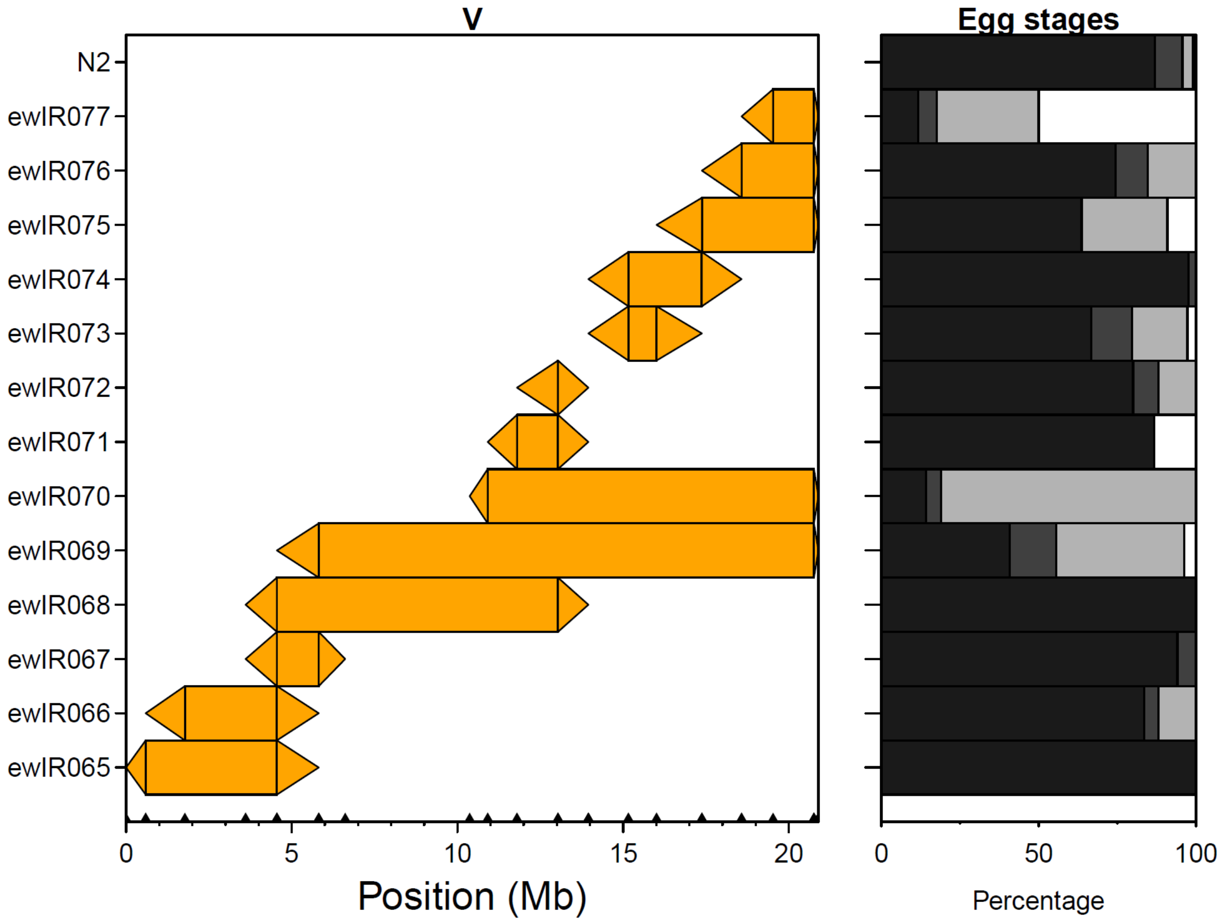

Figure S3 (Continued)

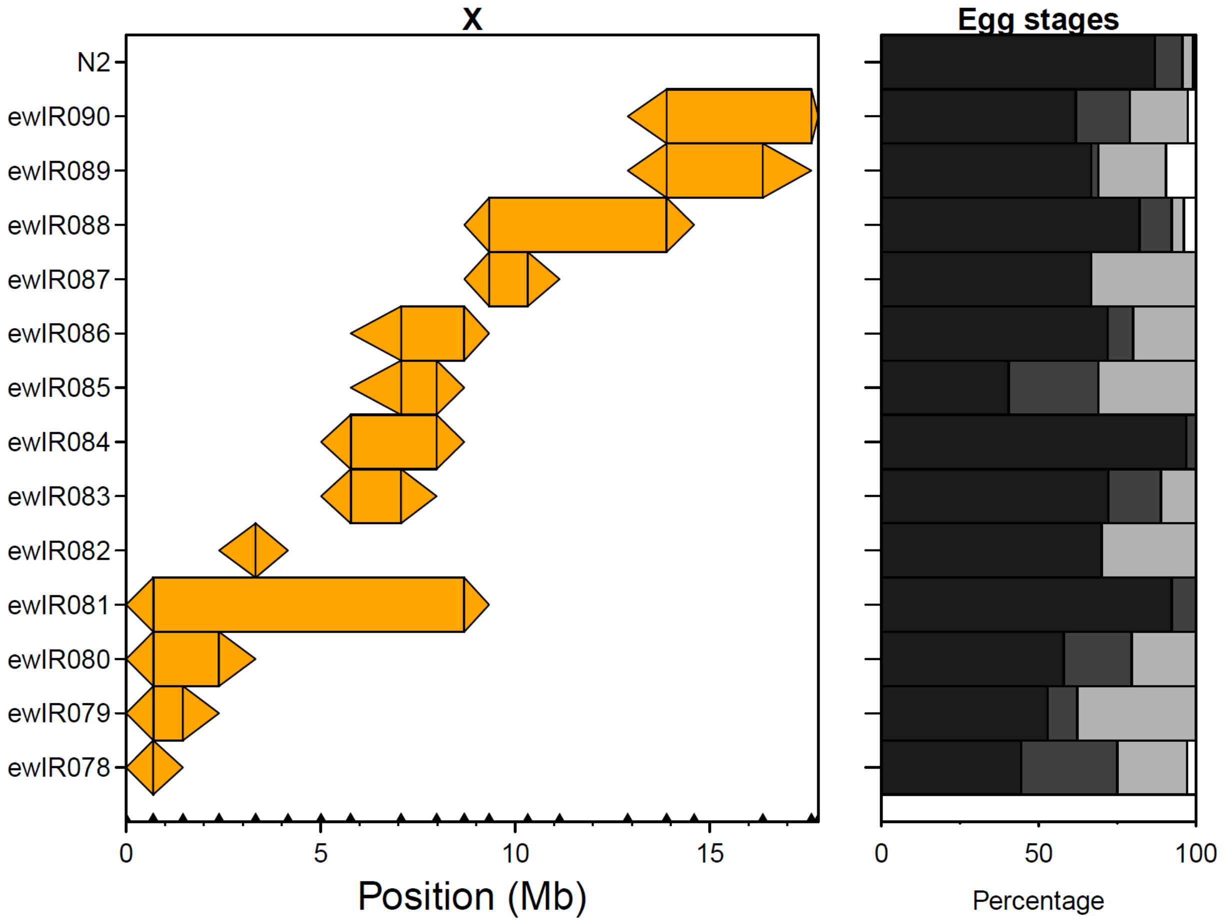

Supplement: Supporting Information [file supp_g3.114.013151_FigureS3.pdf]
