# Supplementary material for: Widespread Genomic Incompatibilities in Caenorhabditis elegans
Source: G3 (Bethesda). 2014 Aug 15;4(10):1813–23. doi: 10.1534/g3.114.013151 (PMC4199689; doi:10.1534/g3.114.013151)
Supplement: Supporting Information [file supp_g3.114.013151_FigureS4.pdf]

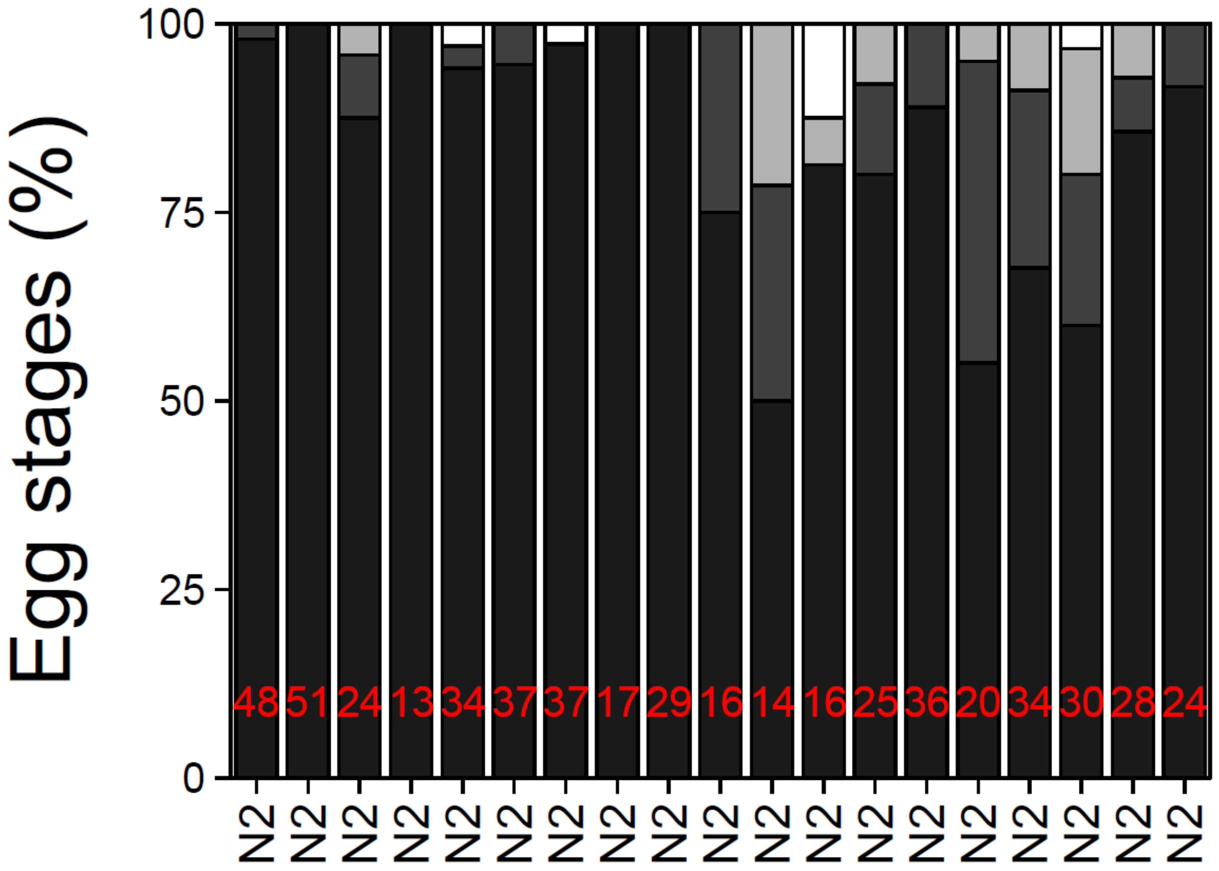

Figure S4 Egg-stages in single N2 dishes used in this paper. The number of eggs measured is indicated in red. Progeny stage distribution is shown as cumulative percentage of total progeny. From dark to light: Stage I, II, III and L1 (in white).
