# Supplementary material for: Widespread Genomic Incompatibilities in Caenorhabditis elegans
Source: G3 (Bethesda). 2014 Aug 15;4(10):1813–23. doi: 10.1534/g3.114.013151 (PMC4199689; doi:10.1534/g3.114.013151)
Supplement: Supporting Information [file supp_g3.114.013151_TableS1.pdf]

Table S1 **IL vs IL Egg-stages. Significance scores in –log10(p)**

ID	Test order	Scores (chi-square test)	N2L	CBL	CBR	N2R	CB_late_stage
1	N2 → 1	1.76					
2	N2 → 2	3.33					
3	N2 → 3	17.51	2818974	3502476	3502476	4338254	+
3	3 → 6	5.17					
3	6 → 7	4.01					
4	N2 → 13	13.13	9569913	10259909	10259909	11085295	+
4	13 → 8	2.82					
4	8 → 5	3.62					
4	5 → 7	1.23					
5	N2 → 6	15.88	2818974	3502476	8654361	9569913	+
6	N2 → 13	13.13	9569913	10259909	10259909	11085295	+
6	13 → 14	12.83	11085295	11085295	11085295	11760179	-
7	N2 → 8	14.34	8654361	9569913	10259909	11085295	+
7	8 → 9	12.02	11085295	11085295	11085295	11760179	-
7	9 → 11	4.56					
7	11 → 12	0.44					
8	N2 → 18	1.18					
8	18 → 17	1.26					
8	17 → 16	0.5					
8	16 → 15	10.26	10259909	11085295	11085295	11760179	+
8	15 → 12	3.0					
9	N2 → 19	0.58					
9	19 → 20	1.33					
9	20 → 21	16.16	2755074	3403575	4147051	4800868	+
9	21 → 22	33.06	4147051	4800868	10414073	11180836	-
10	N2 → 23	1.45					
10	23 → 22	1.21					
11	N2 → 26	2.46					
11	26 → 27	1.54					
12	N2 → 33	4.43					
12	33 → 30	3.94					

12	30 → 28	9.34					
13	N2 → 29	3.68					
13	29 → 30	3.92					
14	N2 → 32	0.38					
15	N2 → 33	4.43					
15	33 → 34	6.97					
15	34 → 35	10.31	5431252	5925983	9308858	10027496	-
15	35 → 38	9.13					
16	N2 → 41	33.67	7998164	8318553	10613119	11341120	+
16	41 → 39	35.77	5925983	6847169	7998164	8318553	-
17	N2 → 40	1.88					
17	40 → 41	38.72	10027496	10613119	10613119	11341120	+
17	41 → 42	26.10	10613119	11341120	11341120	12301725	-
18	N2 → 43	1.26					
18	43 → 44	0.89					
19	N2 → 44	0.16					
20	44 → 38	2.14					
21	N2 → 45	9.89	0	151889	1381409	2288742	+
21	45 → 46	0.16					
21	46 → 47	1.26					
22	N2 → 53	12.90	2288742	3067374	3067374	3920366	+
22	53 → 49	0.32					
22	49 → 48	2.25					
22	48 → 47	0.71					
2	N2 → 49	12.88	1381409	2288742	3067374	3920366	+

3							
23	49 → 50	0.58					
23	50 → 51	4.17					
23	51 → 4001	0.80					
23	4001 → 52	0.80					
24	N2 → 53	12.90	2288742	3067374	3067374	3920366	+
24	53 → 4002,4003,4004	2.23,1.25,1.12					
25	N2 → 59	2.32					
25	59 → 4011	12.72	10909560	11668242	11668242	12748880	+
25	4011 → 4010,56	11.89,21.71	10122930	10909560	10909560	11668242	-
25	4010,56 → 55	6.67,4.10					
25	55 → 54	8.87					
25	54 → 4009,4008,4007	2.13,5.69,9.40					
25	4009,4008,4007 → 4006,4005	4005 vs 4009 12.22					
25	4006,4005 → 4004,4003,4002	NS					
25	4004,4003,4002 → 52	NS					
26	N2 → 56,4010	0.71,2.00					
26	56,4010 → 58	8.57,1.45					
27	N2 → 59	2.32					
27	59 → 60	0.78					
28	N2 → 61	0.19					
29	N2 → 66	2.55					
29	66 → 65	3.88					

30	N2 → 67	0.80					
30	67 → 68	1.39					
31	N2 → 72	0.97					
31	72 → 71	6.62					
31	71 → 68	3.33					
32	N2 →77	26.95	18574593	19525561	20758352	20893784	+
32	77 → 76	21.39	17377158	18574593	18574593	19525561	-
32	76 → 75	4.50					
32	75 → 70	14.74	10368660	10912994	16008404	17377158	+
32	70 → 69	6.89					
33	N2 → 73	2.54					
33	73 → 74	6.60					
34	N2 → 78	8.08					
34	78 → 79	3.50					
34	79 → 80	2.20					
34	80 → 81	7.58					
35	N2 → 83	1.50					
35	83 → 84	5.25					
36	N2 → 85	10.19	5770179	7067019	7982354	8691677	+
36	85 → 84	16.25	5010049	5770179	5770179	7067019	-
37	N2 → 87	7.31					
37	87 → 88	7.78					
38	N2 → 89	5.13					
38	89 → 90	2.83					

IL vs IL Size

ID	Test order	Scores (t-test)	N2L	CBL	CBR	N2R	CB_Size
9	N2 → 19	0.27					
9	19 → 20	0.67					
9	20 → 21	0.29					
9	21 → 22	0.40					
10	N2 → 23	0.46					
10	23 → 22	0.01					
11	N2 → 26	0.59					
11	26 → 27	0.52					
21	N2 → 45	0.10					
21	45 → 46	0.68					
21	46 → 47	0.19					
22	N2 → 53	0.29					
22	53 → 49	0.03					
22	49 → 48	1.11					
22	48 → 47	0.31					
23	N2 → 49	0.42					
23	49 → 50	0.08					
23	50 → 51	0.26					
23	51 → 52	1.30	5819735	6599685	12748880	13667267	-
24	N2 → 53	0.29					
25	N2 → 59	1.45	11668242	12748880	12748880	13667267	+
25	59 → 56	1.08					
25	56 → 55	0.34					
25	55 → 54	1.37	8397264	9102404	9102404	10122930	+
25	54 → 52	1.01					
26	N2 → 56	0.05					
26	56 → 58	1.18					
27	N2 → 59	1.45	11668242	12748880	12748880	13667267	+
27	59 → 60	0.03					
28	N2 → 62	1.64	12748880	13667267	16371991	17084259	-

IL vs IL Lifespan

ID	Test order	Scores (t-test)	N2L	CBL	CBR	N2R	CB_Lifespan
9	N2 → 19	0.07					
9	19 → 20	0.17					
9	20 → 21	0.46					
9	21 → 22	2.69	4147051	4800868	10414073	11180836	+
10	N2 → 23	0.09					
10	23 → 22	1.75	0	176721	2755074	3403575	+

11	N2 → 26	0.96					
11	26 → 27	0.02					
21	N2 → 45	0.20					
21	45 → 46	0.13					
21	46 → 47	0.16					
22	N2 → 53	0.89					
22	53 → 49	0.50					
22	49 → 48	0.24					
22	48 → 47	0.55					
23	N2 → 49	0.24					
23	49 → 50	0.09					
23	50 → 51	1.30	3920366	4991858	5819735	6599685	-
23	51 → 52	0.65					
24	N2 → 53	0.89					
25	N2 → 59	0.38					
25	59 → 56	0.17					
25	56 → 55	0.73					
25	55 → 54	0.26					
25	54 → 52	0.77					
26	N2 → 56	0.54					
26	56 → 58	0.13					
27	N2 → 59	0.38					
27	59 → 60	0.05					
28	N2 → 62	0.54					

IL vs IL Fecundity

ID	Test order	Scores (t-test)	N2L	CBL	CBR	N2R	CB_Fecundity
9	N2 → 19	0.11					
9	19 → 20	0.23					
9	20 → 21	0.53					
9	21 → 22	3.37	4147051	4800868	10414073	11180836	+
10	N2 → 23	0.77					
10	23 → 22	2.87	0	176721	2755074	3403575	+
11	N2 → 26	0.67					
11	26 → 27	0.98					
21	N2 → 45	0.37					
21	45 → 46	0.31					
21	46 → 47	0.62					
22	N2 → 53	0.36					
22	53 → 49	0.20					
22	49 → 48	0.77					
22	48 → 47	0.02					
23	N2 → 49	0.72					
23	49 → 50	0.59					

23	50 → 51	0.84					
23	51 → 52	0.99					
24	N2 → 53	0.36					
25	N2 → 59	0.14					
25	59 → 56	0.01					
25	56 → 55	1.14					
25	55 → 54	0.18					
25	54 → 52	0.58					
26	N2 → 56	0.13					
26	56 → 58	0.95					
27	N2 → 59	0.14					
27	59 → 60	1.34	12748880	13667267	16371991	17084259	-
28	N2 → 62	2.13	12748880	13667267	16371991	17084259	-
